# From data extraction to analysis: a comparative study of ELISE capabilities in scientific literature

**DOI:** 10.3389/frai.2025.1587244

**Published:** 2025-05-12

**Authors:** Maxime Gobin, Muriel Gosnat, Seindé Toure, Lina Faik, Joel Belafa, Antoine Villedieu de Torcy, Florence Armstrong

**Affiliations:** Biolevate, Paris, France

**Keywords:** scientific literature, systematic review, data extraction, AI tool, Elevated LIfe SciencE solution

## Abstract

The exponential growth of scientific literature presents challenges for pharmaceutical, biotechnological, and Medtech industries, particularly in regulatory documentation, clinical research, and systematic reviews. Ensuring accurate data extraction, literature synthesis, and compliance with industry standards require AI tools that not only streamline workflows but also uphold scientific rigor. This study evaluates the performance of AI tools designed for bibliographic review, data extraction, and scientific synthesis, assessing their impact on decision-making, regulatory compliance, and research productivity. The AI tools assessed include general-purpose models like ChatGPT and specialized solutions such as ELISE (Elevated LIfe SciencEs), SciSpace/Typeset, Humata, and Epsilon. The evaluation is based on three main criteria: Extraction, Comprehension, and Analysis with Compliance and Traceability (ECACT) as additional dimensions. Human experts established reference benchmarks, while AI Evaluator models ensure objective performance measurement. The study introduces the ECACT score, a structured metric assessing AI reliability in scientific literature analysis, regulatory reporting and clinical documentation. Results demonstrate that ELISE consistently outperforms other AI tools, excelling in precise data extraction, deep contextual comprehension, and advanced content analysis. ELISE’s ability to generate traceable, well-reasoned insights makes it particularly well-suited for high-stakes applications such as regulatory affairs, clinical trials, and medical documentation, where accuracy, transparency, and compliance are paramount. Unlike other AI tools, ELISE provides expert-level reasoning and explainability, ensuring AI-generated insights align with industry best practices. ChatGPT is efficient in data retrieval but lacks precision in complex analysis, limiting its use in high-stakes decision-making. Epsilon, Humata, and SciSpace/Typeset exhibit moderate performance, with variability affecting their reliability in critical applications. In conclusion, while AI tools such as ELISE enhance literature review, regulatory writing, and clinical data interpretation, human oversight remains essential to validate AI outputs and ensure compliance with scientific and regulatory standards. For pharmaceutical, biotechnological, and Medtech industries, AI integration must strike a balance between automation and expert supervision to maintain data integrity, transparency, and regulatory adherence.

## 1 Introduction

In recent years, the integration of artificial intelligence (AI) into scientific research has significantly transformed academic publishing, drug development, and biomedical innovation. AI-driven literature analysis tools, or AI tools, have become essential, offering groundbreaking capabilities in processing, analyzing, and summarizing large volumes of scientific papers (Agrawal et al., 2024; Danler et al., 2024; Khalifa and Ibrahim, 2024). These tools leverage advanced natural language processing (NLP) techniques to streamline traditionally manual tasks, such as literature reviews and data extraction (Fabiano et al., 2024). The development of sophisticated AI algorithms, fueled by increased computational power and growing data availability, has revolutionized how researchers and industry professionals interact with scientific literature (Zahra et al., 2024).

AI-driven literature analysis tools can be broadly categorized into general-purpose models and specialized scientific models, each serving distinct roles in research. General-purpose AI models, such as OpenAI’s ChatGPT, are designed for versatility, able to handle a wide array of tasks across various domains. These models excel in summarizing and interpreting general information, offering researchers a flexible tool for diverse inquiries (Ning et al., 2024). In contrast, specialized tools like ELISE (the tool we developed at Biolevate), SciSpace (ex-Typeset), Humata, and Epsilon are tailored to meet the specific needs of scientific and biomedical research, including pharmaceutical, biotech and Medtech applications (Zahra et al., 2024; Tsirmpas et al., 2024). These applications focus on precision and relevance, employing advanced NLP techniques to extract and analyze data with a high level of specificity for regulated and highly technical fields. While general-purpose tools provide broad applicability, specialized tools ensure accuracy and domain relevance, crucial for rigorous academic inquiry.

More specifically, ChatGPT excels in conversational interactions, aiding in literature reviews and hypothesis generation (Khalifa and Ibrahim, 2024). SciSpace/Typeset enhances literature exploration with intuitive interfaces and robust search capabilities (SciSpace, 2024). Humata focuses on AI-driven text analysis, efficiently summarizing large volumes of data (Humata, 2024). Epsilon identifies research gaps, suggesting innovative directions (Epsilon, 2024). Meanwhile, ELISE advances document analysis with sophisticated NLP and data extraction techniques, addressing the growing need for efficient data management in research (ELISE, 2024).

Together, these AI tools enhance research quality and efficiency, facilitating a more comprehensive understanding of complex scientific literature and accelerating the pace of discovery by reducing the time required for knowledge synthesis (Borah et al., 2017; Chakraborty et al., 2024; Mehta et al., 2024). They support key industry processes, from early-stage drug discovery to regulatory documentation preparation, helping companies navigate vast and evolving scientific landscape (Agrawal et al., 2024). For pharmaceutical, biotech, and Medtech companies, AI tools can improve literature surveillance, support regulatory submissions (e.g., EMA/FDA filings), and optimize knowledge management for evidence-based decision-making.

Despite these advantages, integrating AI into scientific research is not without challenges, particularly in ensuring the reliability and consistency of AI-generated insights. A significant issue is the variability in AI-generated responses, which stems from the diverse methodologies and algorithms employed by different tools (Bolaños et al., 2024). This inconsistency complicates the standardization of evaluations, as outputs can vary in quality and relevance. Additionally, the lack of traceability in AI-generated insights raises concerns about the validity of interpretations, particularly in fields requiring high precision, such as biomedical and clinical research (Agrawal et al., 2024; Danler et al., 2024). Furthermore, the absence of a standardized framework for evaluating AI tools poses a threat to research reliability and validity (Ning et al., 2024; Sharma and Ruikar, 2024).

Therefore, there is a pressing need for rigorous and standardized evaluations to ensure that AI tools contribute effectively and reliably to scientific research, safeguarding the rigor and integrity of research outcomes.

This study aims to provide the scientific community and industry stakeholders with a clearer understanding of the potential and limitations of AI-driven literature analysis technologies. It also introduces a structured and independent methodology based on three main criteria (Extraction, Comprehension, and Analysis), to evaluate these applications, including ELISE, a novel AI tool integrating NLP and retrieval strategies to enhance workflows, improve regulatory compliance and optimize R&D processes for pharma, biotech and Medtech organizations.

## 2 Methods

### 2.1 Selected articles

To ensure a comprehensive and exhaustive evaluation, a diverse selection of scientific articles was chosen, covering different disciplines and study types. The selected articles are detailed in Table 1.

**Table 1:** Overview of selected articles, including title, type, discipline, and citation.

Article no.	Title	Type	Discipline	Citation
1	Functions of double-stranded RNA-binding domains in nucleocytoplasmic transport	Experimental study	Biology	Banerjee and Barraud (2014)
2	Protein dimerization via Tyr residues: highlight of a Slow Process with co-existence of numerous intermediates and final products	Experimental study	Chemistry	Gatin et al. (2022)
3	Nivolumab-AVD in Advanced Stage Classic Hodgkin Lymphoma	Experimental study	Medicine	Herrera et al. (2024)
4	Extracellular HMGB1 blockade inhibits tumor growth through profoundly remodeling immune microenvironment and enhances checkpoint inhibitor-based immunotherapy	Experimental study	Biology	Hubert et al. (2021)
5	Impact of COVID-19 on Mental Health in Adolescents: A Systematic Review	Systematic review	Medicine	Jones et al. (2021)
6	Le système de surveillance des anomalies congénitales de l’Alberta: compte rendu des données sur 40 ans avec prévalence et tendances de certaines anomalies congénitales entre 1997 et 2019	Quantitative research - French version	Public health	Lowry et al. (2023a)
7	The Alberta Congenital Anomalies Surveillance System: a 40-year review with prevalence and trends for selected congenital anomalies, 1997–2019	Quantitative research - English version	Public health	Lowry et al. (2023b)
8	Addition of four doses of rituximab to standard induction chemotherapy in adult patients with precursor B-cell acute lymphoblastic leukaemia (UKALL14): a phase 3, multicentre, randomized controlled trial	Experimental study	Medicine	Marks et al. (2022)
9	Research Progresses and Applications of Knowledge Graph Embedding Technique in Chemistry	Theoretical study	Chemistry	Wang et al. (2024)

The current selection emphasized experimental and applied biomedical literature to stress-test AI tools in high-rigor contexts. Future work will include broader disciplinary representation to assess generalizability. This initial dataset includes articles in both English and French, allowing us to test cross-lingual consistency of the ECACT scoring framework. However, broader validation across other languages and domains remains a priority for future work.

The sample was restricted to nine articles to allow for a controlled proof-of-concept analysis. This scale enabled full evaluation across five criteria and three evaluators per article. However, broader validation will require expansion to larger datasets.

### 2.2 AI models used for evaluation

#### 2.2.1 AI tools

Among the most popular, several AI-driven literature analysis tools were selected based on their ability to extract, comprehend, and analyze scientific content. Table 2A summarizes their main characteristics.

**Table 2:** Overview of selected AI tools **(A)** and AI evaluator models **(B)**.

A				
AI tools	Provider	Release date	Citations capacity	Highlighting capacity
ChatGPT (GPT-4o)	OpenAI	November 2024	No	No
ELISE 2.0	Biolevate	December 2024	Yes	Yes
Epsilon 2.5	Epsilon	April 2024	Yes	Yes
Humata	Tilda technologies	November 2024	Yes	Yes
SciSpace/Typeset 1.4.12	SciSpace (ex-Typeset)	November 2024	Yes	Yes

B			
AI evaluator models	Company	Launching date	Reasoning model
Claude 3.5 Sonnet	Anthropic	June 2024	NO
GPT-4o	OpenAI	May 2024	NO
O1 Preview	OpenAI	December 2024	YES

#### 2.2.2 AI evaluator models

To assess the performance of the AI tools, independent AI Evaluator models were used, as described in Table 2B. To ensure a neutral and reproducible evaluation process, we selected three independent, high-performing AI models to act as evaluators: GPT-4o (OpenAI), Claude 3.5 Sonnet (Anthropic), and o1 Preview (OpenAI).

These models were intentionally chosen from distinct technical ecosystems and were not used at any stage in the development, training, or fine-tuning of the ELISE engine. This separation was critical to avoid any potential overlap or bias arising from shared components.

The selected models are known for their advanced reasoning and summarization capabilities, making them suitable for structured judgment across multiple scientific domains. Their inclusion was further motivated by public availability, multilingual support, and architectural diversity (e.g., different training corpora, prompting behaviors, and instruction-following styles).

Each model performed evaluations independently, using randomized prompts under both anonymized and non-anonymized conditions, and their scores were subsequently averaged to control for evaluator-specific bias.

#### 2.2.3 Bias identification and model selection

To identify potential biases in AI-generated evaluations, we conducted a control assessment using Article 5 (Jones et al., 2021) on the Comprehension and Analysis criteria.

- AI tools were evaluated under two conditions: with their identities revealed and anonymized.
- A comparison of the scoring results under both conditions allowed us to assess any bias in evaluator decisions.

To ensure transparency, AI tool responses were evaluated under both identified and anonymized conditions. For anonymized evaluations, tool names were replaced with neutral codes (Tool A, Tool B, etc.), and the order of responses was randomized. The evaluators were not informed of the anonymization. This protocol was repeated for all three evaluator models, and scoring results were then compared across both conditions to assess potential bias. Supplementary Table 1 presents the comparative results.

### 2.3 Evaluation criteria

To objectively assess the performance of AI tools, three primary criteria were selected: Extraction, Comprehension, and Analysis. Each criterion was evaluated through a structured questionnaire described in Table 3.

**Table 3:** Criteria and evaluation questions.

Criteria	Questions	Guidelines
Extraction	Author’s names	
	Study sponsor	
	Year of publication	
	Journal’s name	
	DOI number	
	Product evaluated in the study	
	Type of study	
	Indication of the study	
	Eligibility criteria	
Comprehension	Objectives	(in two sentences)
	Risk of bias	(factually, in one sentence)
	Methodology	(only from data’s in the document: present in one sentence the global methodology of the study and if it described, present in one sentence different methodologies used in the study)
	Main results	(in one sentence and for each part of the study results section, present the conclusion)
	Secondary results	(in one sentence and for each sub-part of the study results section, if any, present the conclusion)
	Conclusion of the study	(in two sentences)
	Summary of the abstract	(in three sentences)
	Summary of the study	(one sentence for each main section of the study: introduction, method, results, discussions, conclusion)
Analysis	Interpretation of the mains results	(in one sentence for each main result, discuss/enhance results)
	Limitations of the study	(in one sentence for each limitation)
	Three specific questions	(only from data of the document, in one sentence)
	Prospects for the future	(only from data of the document, in one sentence)

To limit subjectivity in open-ended tasks such as summarization, each question was paired with an expected response profile. Evaluators followed a standardized 10-point rubric (see Supplementary Table 3), and were prompted to assess semantic fidelity, relevance, and completeness rather than superficial similarity. For comparison-based judgments, evaluators used structured prompts to evaluate content quality and alignment with the source article.

### 2.4 Evaluation protocol and scoring

Before evaluation, the questions listed in Table 3 were submitted to each AI tool for every article and every evaluation criterion. The queries were conducted between October 2024 and January 2025, and all responses were collected. The full evaluation process was carried out in January 2025.

#### 2.4.1 Scoring methodology

##### 2.4.1.1 Extraction criterion

**Figure 1:**
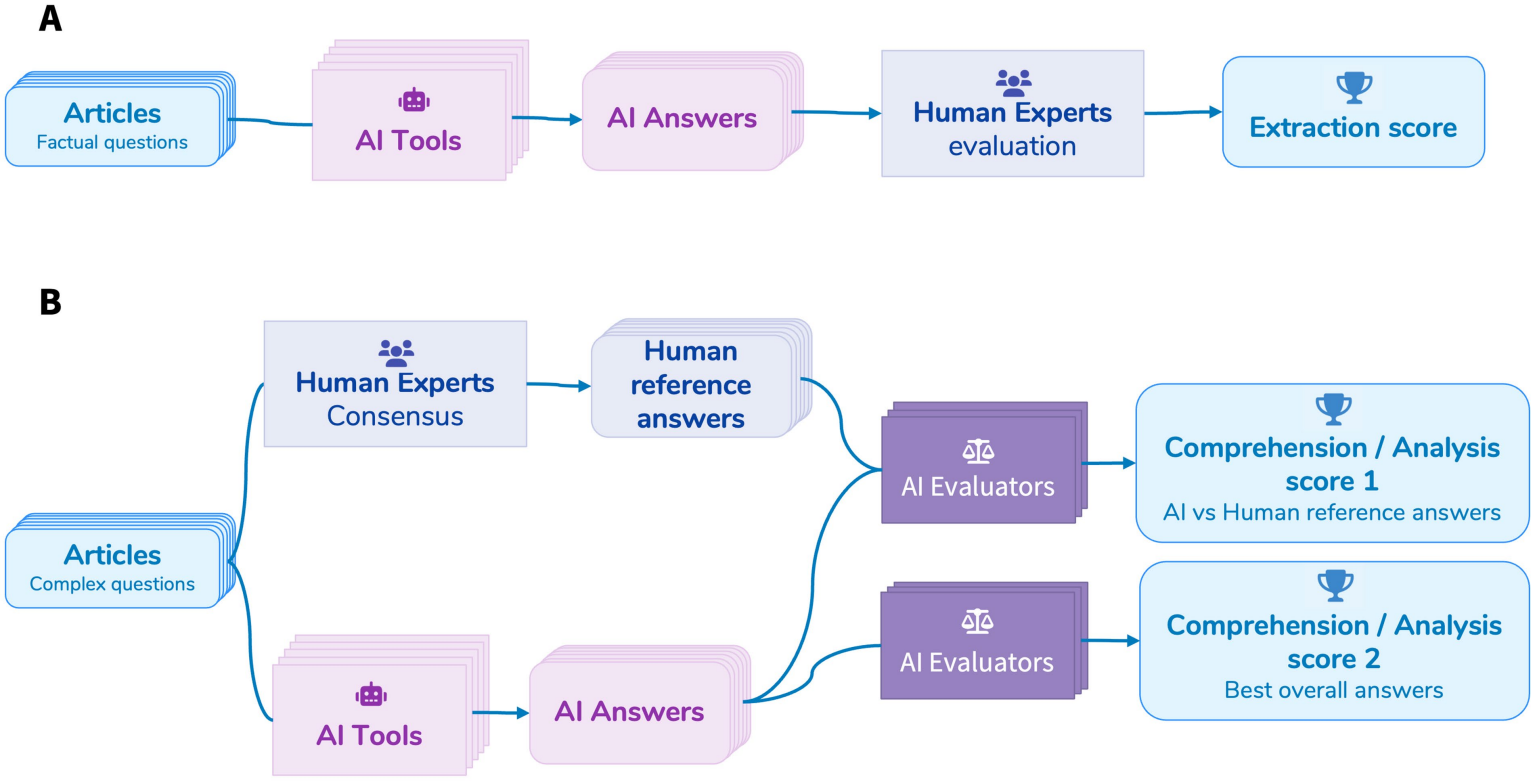
Extraction **(A)** and comprehension/analysis **(B)** evaluation protocol.

- Each response was manually checked by a human reviewer against expected data.
- Each answer was scored out of 1, with partial credit assigned proportionally based on the number of correctly retrieved elements. For instance, if an article was authored by three individuals and the AI tool retrieved only one name, the response received a score of 0.33/1. Similarly, if a study had four different sponsors but only two were correctly identified, the response was scored 0.5/1.
- For each article and each AI tool, a percentage score was calculated by summing the points obtained across all questions (see Figure 1A).

##### 2.4.1.2 Comprehension and analysis criteria

- First, three independent human experts answered the questions, without knowing the answers of the AIs, to establish a reference baseline.
- Second, two different evaluations were performed using automated evaluation prompts provided in Supplementary Figures 1A,B:

- Each AI Evaluator model (presented in Table 2B) assessed AI-generated responses against human-defined reference answers.
- AI Evaluator models independently assessed the responses based solely on document content, without human answers, to evaluate autonomous interpretability of AI-generated content.

- Each response was scored out of 10 by the AI Evaluator models and justified by them.
- For each article and each AI tool, a percentage score was calculated by summing the points obtained across all questions (see Figure 1B).

##### 2.4.1.3 Final performance assessment

- To assess global performance, the average score obtained across all evaluated articles was calculated for each AI tool.
- Additionally, the standard deviation of scores was determined for each AI tool to measure variability in performance across different articles.

##### 2.4.1.4 Compliance and traceability scoring

- *Compliance score (0–10)*: evaluates if AI responses strictly adhered to the document content and followed the required guidelines.
- *Traceability score (0–10)*: assesses whether AI tools correctly highlighted the relevant sections used in their answers.

##### 2.4.1.5 Final ECACT score calculation

A weighed global score (ECACT score) proposition was created to reflect the importance of each evaluation criterion:


$$\text{ECACT}=(E*1.0)+(C*1.5)+(A*2)+(C_{\text{comp}}*1.5)+(T*2.0)$$

Where E = Extraction, C = Comprehension, A = Analysis, C_comp_ = Compliance, and T = Traceability, leading to a final score out of 80 points.The weighting system was designed to reflect the relative importance of each criterion in regulated scientific contexts. Analysis and Traceability were prioritized (×2.0) due to their implications for interpretability and compliance, particularly in biomedical and regulatory workflows. A sensitivity analysis exploring alternative weighting schemes is provided in Supplementary Table 2.

### 2.5 Statistical analysis

To ensure statistical robustness and objectivity in comparing the performance of AI tools, a comprehensive statistical framework was applied. All statistical analyses were conducted by a trained statistician (LF, co-author).

First, we performed a one-way ANOVA for each evaluation criterion (Extraction, Comprehension, Analysis) to determine whether differences in performance scores between AI tools were statistically significant. The assumption of homogeneity of variances was assessed using Levene’s test, which evaluates whether the variances across groups are equal. A non-significant result (*p* > 0.05) confirmed that the assumption was met, allowing the use of standard ANOVA procedures.

Second, a two-way ANOVA was carried out to investigate both the main effects and their interaction effects between AI tool identity and evaluation criteria, thereby assessing whether certain models performed differently depending on the evaluation criterion.

Third, we applied *post-hoc* Tukey Honestly Significant Difference (HSD) tests to identify which pairs of AI tools showed statistically significant differences. This test was chosen for its robustness to multiple comparisons and its suitability for evaluating grouped means.

Additionally, to support transparency and explore the robustness of the ECACT scoring framework, a sensitivity analysis was conducted. Alternative weighting schemes (e.g., equal weights, compliance-prioritized, comprehension-focused) were applied to assess how different weight configurations impacted the final AI tool rankings. Results showed that while some mid-ranking positions shifted, the top-performing (ELISE) and lowest-performing tools remained consistent across weighting conditions (see Supplementary Table 2 and Supplementary Figure 4).

All analyses were performed using Python version 3.11.9 and validated independently (statsmodels v.0.14.4 and scipy v.1.14.1 libraries). A *p*-value threshold of 0.05 was used for significance across all tests.

The full evaluation dataset, question prompts, and scoring rubrics are available in the accompanying GitHub repository.^1^

## 3 Results

To provide the scientific community with a clearer understanding of the capabilities and limitations of AI-driven literature analysis applications, we developed a structured evaluation methodology based on three key criteria (Extraction, Comprehension and Analysis). Each of these criteria plays a critical role in assessing the overall effectiveness of AI tools in scientific research.

The evaluation process was conducted by analyzing the responses provided by each AI tool to a predefined set of questions (Table 3) across multiple scientific articles (Table 1). The articles were selected to represent a diverse range of disciplines and study types, ensuring a broad assessment of AI tool performance in different editorial contexts. Additionally, two equivalent articles (one in French and the other in English) were included in the dataset to investigate potential variations in AI performance due to language differences.

### 3.1 Extraction performance

The first evaluation criterion, Extraction, assessed the ability of AI tools to accurately identify and retrieve key bibliographic elements such as author names, publication dates, study types, and other fundamental metadata. These elements are essential for organizing, referencing, and citing scientific work. The questions related to this criterion and their expected responses are detailed in Table 3, with answers consisting exclusively of factual data. For each article and each question, the expected number of correct data points was predefined and AI-generated responses were manually evaluated for completeness and accuracy. Each response was assigned a score of 1 point, with proportional credit awarded when only a subset of the expected data was correctly retrieved (*data not shown*). Then a percentage score was calculated by summing the points obtained across all questions for each article and a global score is obtained for each AI tool.

**Figure 2:**
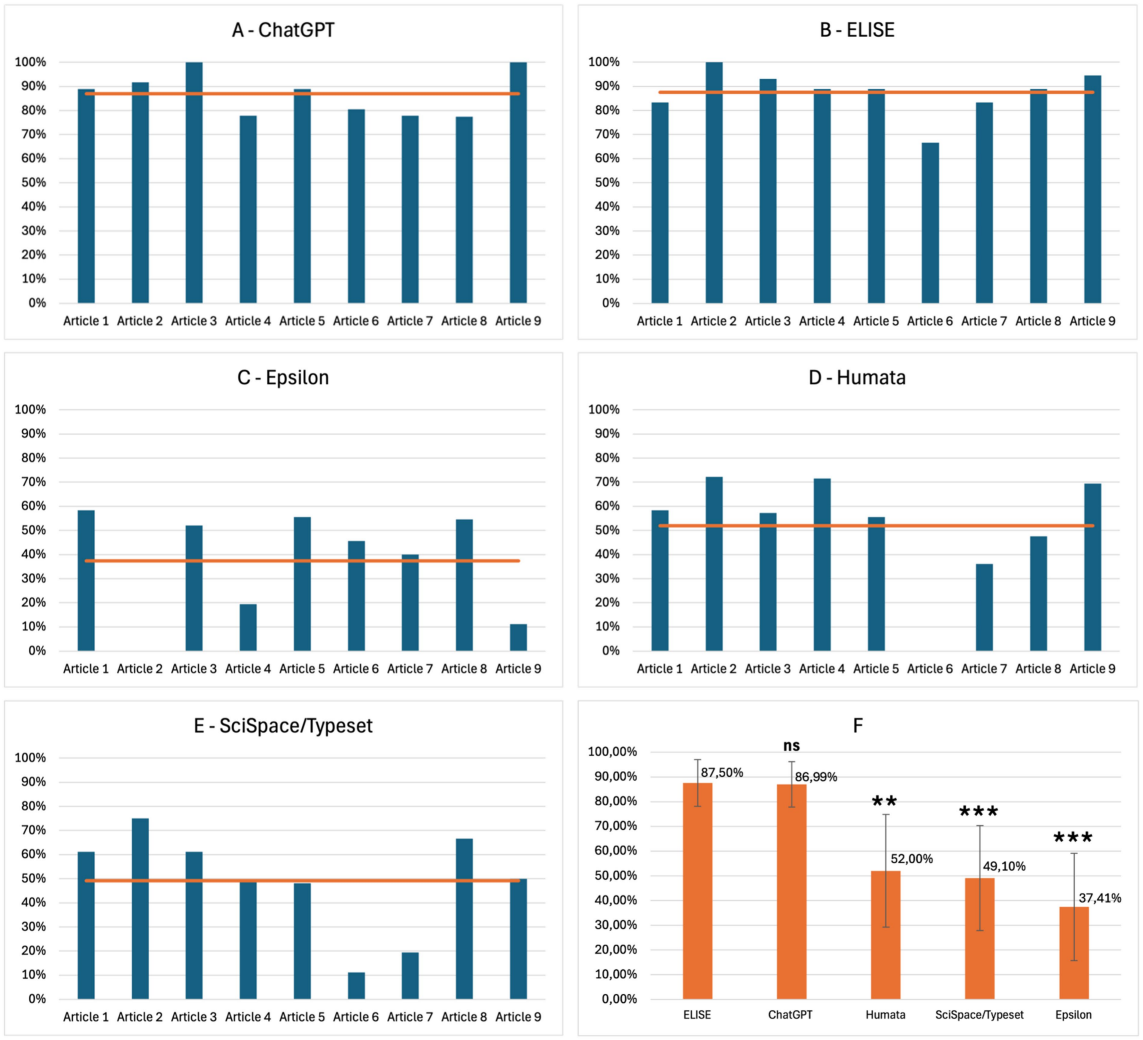
Extraction performance of AI tools - ELISE and ChatGPT are the most effective tools for extracting standard metadata compared to common AI specialized tools. Results of AI tools evaluations for the Extraction criterion, presented by article: ChatGPT **(A)**, ELISE **(B)**, Epsilon **(C)**, Humata **(D)**, SciSpace/typeset **(E)** and global average across all AI tools **(F)**. Statistically significant differences with ELISE are marked with asterisks: ns = not significant (*p* ≥ 0.05), ** = very significant (*p* ≤ 0.01) and *** = highly significant (*p* ≤ 0.001).

The results of the Extraction evaluation are presented in Figure 2, illustrating the performance of each AI tool across different articles as well as the global average performance. ChatGPT (Figure 2A) and ELISE (Figure 2B) demonstrate the highest extraction efficiency, consistently achieving scores above 80%, with a minimal variation across articles. In contrast, Epsilon (Figure 2C), Humata (Figure 2D), and SciSpace/Typeset (Figure 2E) exhibited more variable performance, generally ranging between 60 and 70%, with significant fluctuations depending on the articles. The global performance average (Figure 2F) demonstrates that ELISE (87.50%) and ChatGPT (86.99%) were the most effective tools in extracting standard metadata, significantly outperforming Humata (52.00%), SciSpace/Typeset (49.10%) and Epsilon (37.41%). Statistical analysis revealed significant differences, with ELISE demonstrating a superior performance compared to Humata (p£0.01), SciSpace/Typeset (p£0.001), and Epsilon (p£0.001).

### 3.2 Selection of evaluation models

To further assess the performance of AI tools beyond simple data extraction, we focused on two complex evaluation criteria: Comprehension and Analysis. These criteria require structured, explanatory and context-aware answers, which introduce significant variability in both quality and quantity. Such complexity makes human evaluation challenging, as scoring responses may be influenced by subjectivity and cognitive biases. To mitigate these risks, we established a set of calibrated guidelines (see Table 3) and opted for an AI-based evaluation approach.

A multi-model, independent evaluation was conducted to systematically assess AI tools performance on the Comprehension and Analysis criteria. To ensure robustness, a single reference article (Article 6 - Jones et al., 2021) was used in this evaluation. The primary objective was to determine whether AI Evaluator models introduced biases when grading responses, particularly by comparing identified and anonymized AI tool responses.

**Figure 3:**
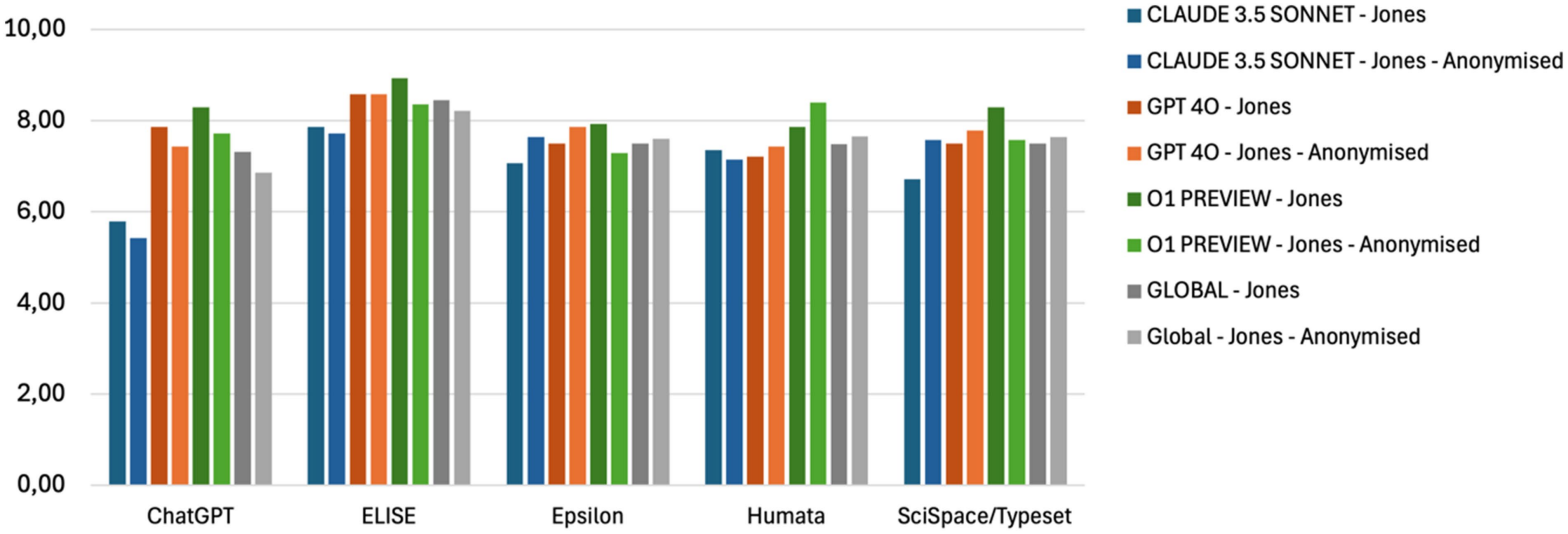
A multi-model evaluation ensuring unbiased AI scoring - Global average of AI tools’ evaluation by AI Evaluator models (Claude 3.5 Sonnet, GPT-4o, o1 Preview and the average global score (Global) for the Comprehension and Analysis criteria applied to Article 5 (Jones et al., 2021). Comparison between identified and anonymized AI tool response.

The evaluation was conducted using three AI Evaluator models: Claude 3.5 Sonnet, GPT-4o, and o1 Preview (Table 2B). The responses from AI tools were processed separately by each evaluator, and their scores were then averaged (Figure 3).

Results indicate minimal variation between evaluations of identified and anonymized answers, demonstrating that the AI Evaluator models were not influenced by the identity of the AI tools being assessed. This consistency supports the reliability and objectivity of the evaluation framework.

The comparison between identified and anonymized scoring (see Supplementary Figure 1) revealed minimal variation for most tools, confirming that AI Evaluator models were not significantly influenced by tool identities. This supports the robustness and neutrality of the evaluation framework.

However, one notable discrepancy was observed in the evaluation of ChatGPT’s responses, where the scores provided by Claude 3.5 Sonnet diverged from those of GPT-4o and o1 Preview. To maintain fairness and accuracy in scoring, as well as to ensure a balanced evaluation, the final assessment of AI tools was determined by incorporating the average results from all three AI Evaluator models. This approach minimizes potential biases and ensures that the final evaluation reflects a comprehensive and standardized assessment of AI-driven comprehension and analysis capabilities.

### 3.3 Comprehension

The second evaluation criterion, Comprehension, aimed to assess the AI tools’ ability to interpret and structure key arguments, conclusions and methodological aspects of scientific articles. This criterion is critical for determining how well AI models can process complex scientific content and provide accurate and coherent summaries.

**Figure 4:**
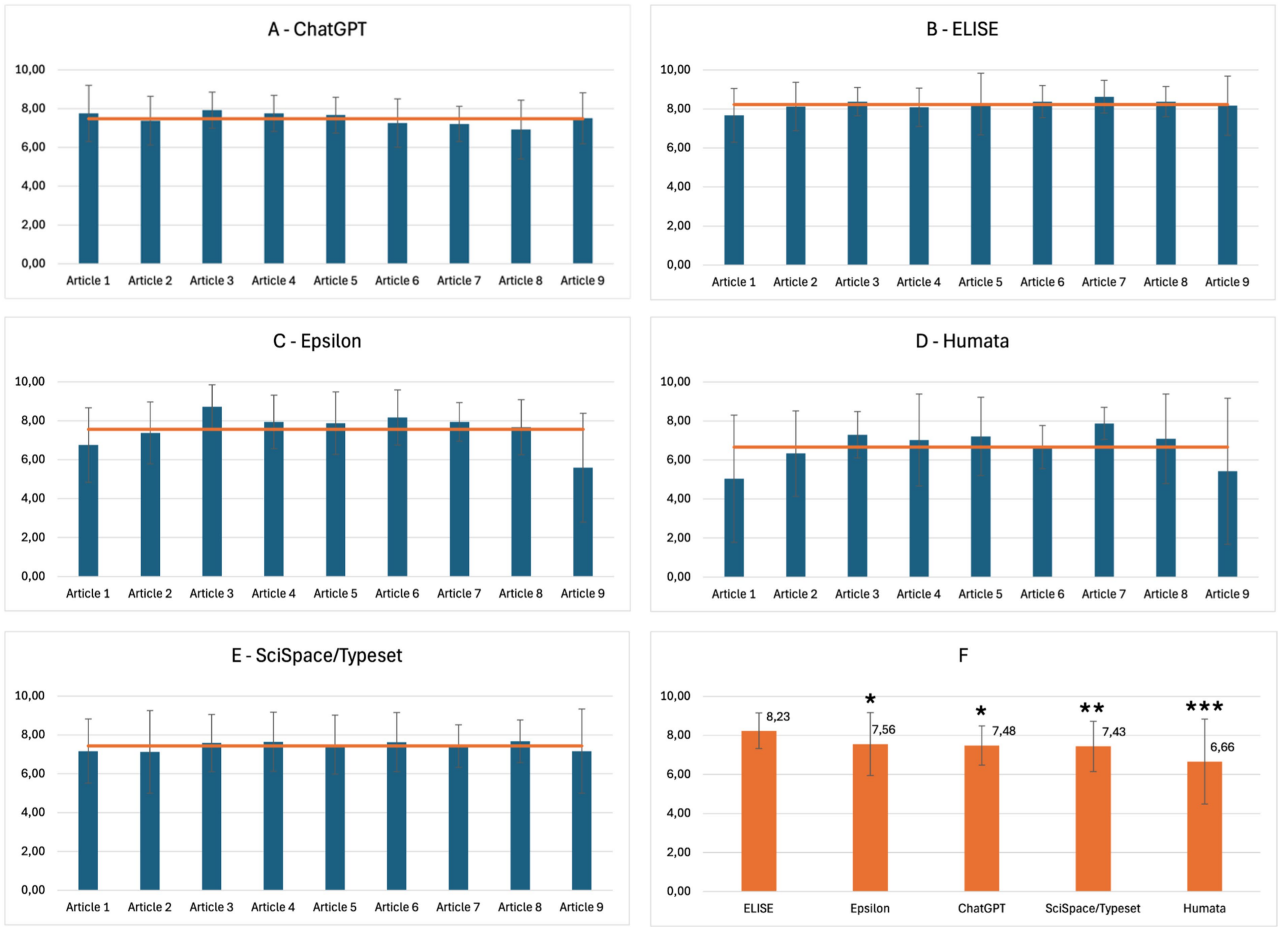
ELISE and Epsilon outperform ChatGPT, Humata, Scispace/Typeset for scientific comprehension. Overall results of AI tools evaluation for the Comprehension criteria: ChatGPT **(A)**, ELISE **(B)**, Epsilon **(C)**, Humata **(D)**, SciSpace/Typeset **(E)**, and Global average of AI tools evaluation **(F)**. Statistically significant differences with ELISE are marked with asterisks: * = significant (*p* ≤ 0.05), ** = very significant (*p* ≤ 0.01) and *** = highly significant (*p* ≤ 0.001).

To ensure an objective evaluation, a reference answer set was established by a panel of three human experts, defining expected responses for each question in every article. Then, the AI-generated responses were assessed by AI Evaluator models, which compared them to the human-defined references. Each response was scored on a 10-point scale, with justifications provided by the AI Evaluator models. The scores obtained for each AI tool, AI Evaluator model, and article were averaged (*data not shown*). The overall final score for each AI tool and article were averaged across the different evaluators and the results are presented in Figure 4.

ELISE (Figure 4B) demonstrated the highest performance in Comprehension, with scores consistently exceeding 8.0, highlighting its ability to process and synthesize scientific information effectively. In contrast, Epsilon (Figure 4C), ChatGPT (Figure 4A) and SciSpace/Typeset (Figure 4E) exhibited moderate performance, with scores ranging between 7.0 and 8.0. Humata (Figure 4D) displayed greater variability with scores fluctuating between 5.0 and 8.0, indicating inconsistencies in its comprehension capabilities. The global evaluation (Figure 4F) demonstrated that ELISE and Epsilon outperformed ChatGPT, Humata, and SciSpace/Typeset with final scores of: 8.23, 7.56, 7.48, 7.46 and 6.66, respectively. Statistical analysis demonstrated significant differences, reinforcing ELISE’s superior comprehension capabilities compared to all other models.

### 3.4 Analysis

The final evaluation criterion, Analysis, focused on assessing each AI tool’s ability to engage in critical reasoning, summarize key findings, identify study limitations, and generate meaningful insights. This criterion is essential for determining whether AI models can go beyond simple text extraction and comprehension to produce deeper, more structured interpretation of scientific content.

**Figure 5:**
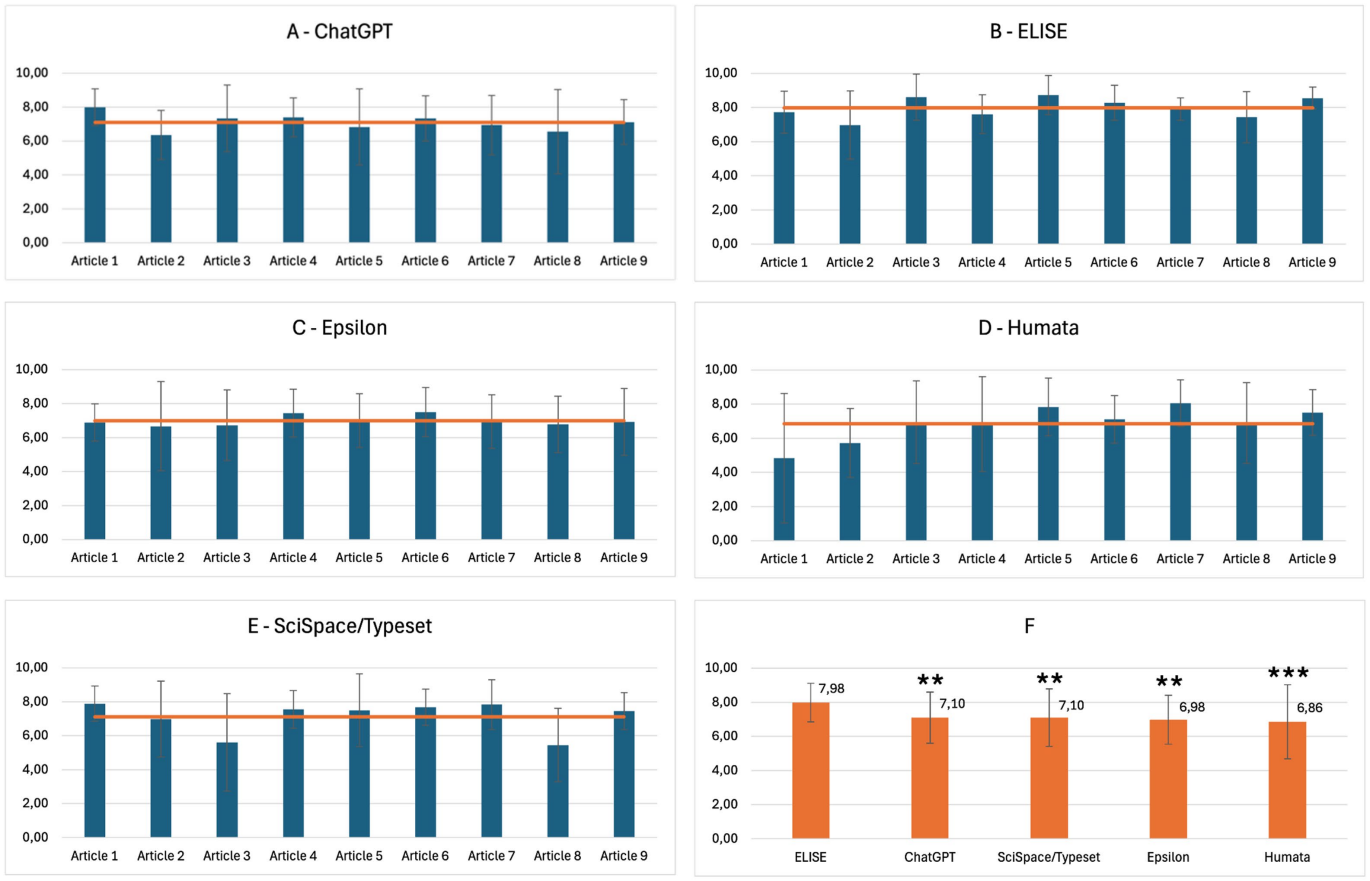
ELISE demonstrates superior analytical capabilities compared to ChatGPT and specialized AI tools. Overall results of AI tools evaluations for the Analysis criterion: ChatGPT **(A)**, ELISE **(B)**, Epsilon **(C)**, Humata **(D)**, SciSpace/Typeset **(E)** and global average of AI tool evaluations (Global). Statistically significant differences with ELISE are marked with asterisks: ** = very significant (*p* ≤ 0.01) and *** = highly significant (*p* ≤ 0.001).

Following the same evaluation methodology used for Comprehension, AI Evaluator models assessed AI-generated responses based on pre-defined human expert answers. Each response was rated on a 10-point scale. The scores obtained for each AI tool, AI Evaluator model, and article were averages (*data not shown*). The overall final score for each AI tool and article were averaged across the different evaluators and the results are presented in Figure 5.

ELISE (Figure 5B) achieved the highest Analysis performance, with scores ranging between 7.0 and 9.0, demonstrating strong critical reasoning capabilities. In contrast, ChatGPT (Figure 5A), SciSpace/Typeset (Figure 5E), Epsilon (Figure 5C) and Humata (Figure 5D), exhibited lower efficiency, with scores fluctuating between 4.5 and 8.0. Greater performance variability was observed in Humata, SciSpace/Typeset and ChatGPT show greater variability in performance compared to Epsilon and ELISE, suggesting inconsistencies in their ability to generate structured and insightful interpretations.

The global performance evaluation (Figure 5F) demonstrated ELISE’s superior analytical capabilities, with a final score of 7.98, significantly surpassing ChatGPT (7.10), SciSpace/Typeset (7.10), Epsilon (6.98) and Humata (6.86). Statistical analyses demonstrated significant differences, reinforcing ELISE’s effectiveness in analyzing scientific content compared to other models.

### 3.5 Language change and AI tools efficiency

**Figure 6:**
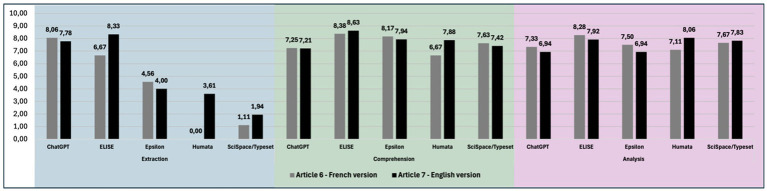
Language change can lead to variations in AI tools responses. Overall results of AI tools (ChatGPT, ELISE, Epsilon, Humata and SciSpace/Typeset) for the extraction, comprehension and analysis criteria comparing performance across French and English articles.

To evaluate the impact of language on AI tools performances, we assessed their ability to process identical articles written in French and English (Article 6 and Article 7 - Lowry et al., 2023a). The evaluation was conducted using the Extraction, Comprehension, and Analysis criteria, and the results are presented in Figure 6.

Performance scores for the Extraction criterion revealed a greater variability across languages. ChatGPT achieved 8.06 in French and 7.78 in English, ELISE 6.67 and 8.33, Epsilon 4.56 and 4.00, Humata 0.00 and 3.61, and SciSpace/Typeset 1.11 and 1.94, respectively. These results suggest that ChatGPT and Epsilon maintained stable performance across both languages, while Humata and ELISE exhibited better results in English.

For Comprehension and Analysis criteria, performance variability across languages was minimal for all AI tools, with no strong preference for one language over the other, except for Humata, which consistently performed better in English. The comprehension scores were 7.25 and 7.21 for ChatGPT, 8.38 and 8.63 for ELISE, 8.17 and 7.94 for Epsilon, 6.67 and 7.88 for Humata, 7.63 and 7.42 for SciSpace/Typeset. The analysis criterion followed a similar trend with 7.33 and 6.94 for ChatGPT, 8.28 and 7.92 for ELISE, 7.50 and 6.94 for Epsilon, 7.11 and 8.06 for Humata, 7.67 and 7.83 for SciSpace/Typeset.

These findings highlight that language influences Extraction performance more than Comprehension and Analysis. While some AI tools perform equally well across languages, others exhibit discrepancies, particularly in data retrieval tasks, emphasizing the need for further linguistic adaptation in AI-driven scientific analysis.

### 3.6 Human expertise and AI tools

**Figure 7:**
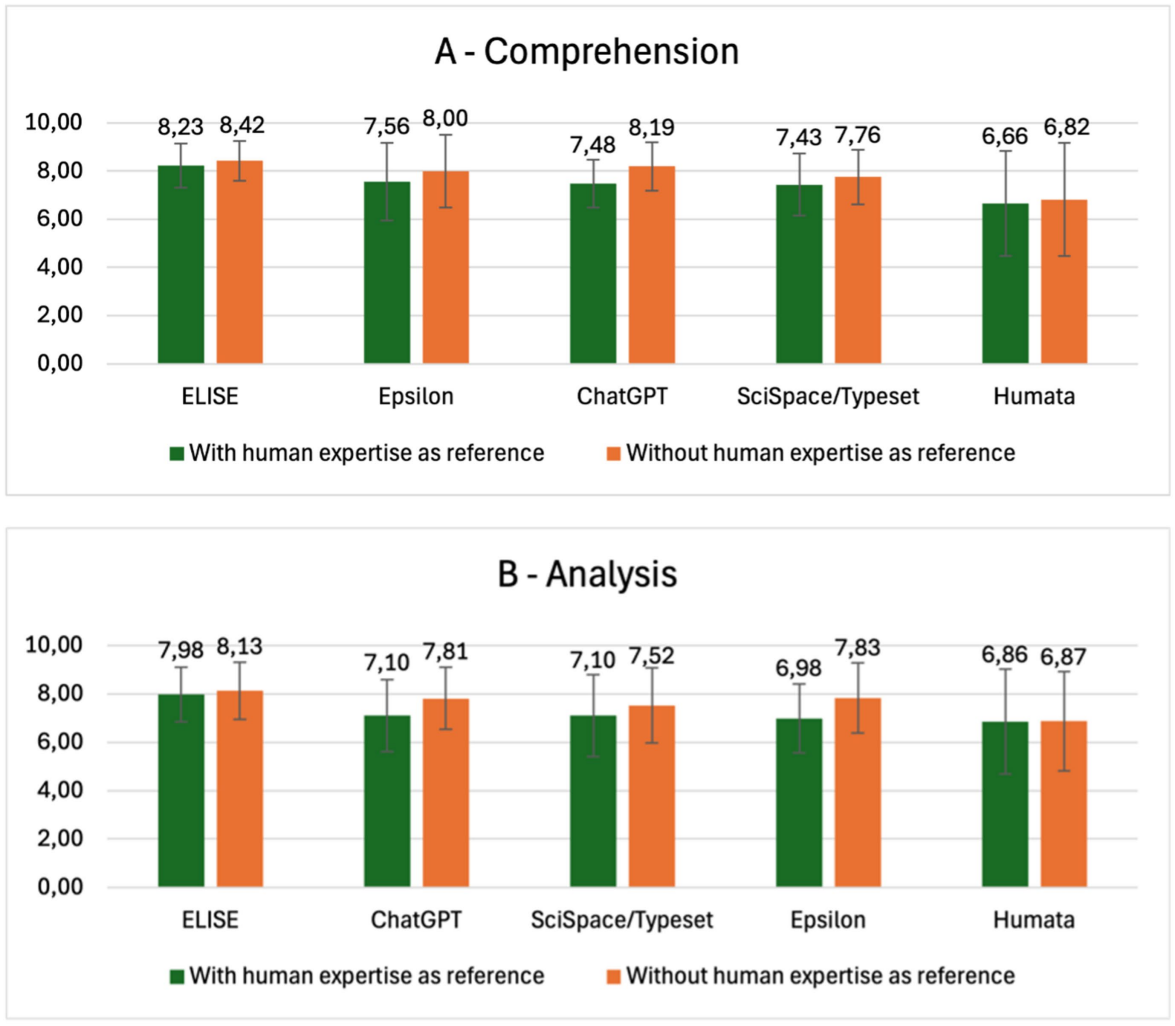
ELISE’s responses align more closely with human expertise than other AI tools. Comparison of AI tool performance in the comprehension **(A)** and analysis **(B)** criteria, evaluated with and without human reference answers.

To further examine AI tools’ capabilities, we assessed their performance in Comprehension and Analysis criteria without providing human-validated reference answers. The goal was to evaluate how AI tools perform autonomously when interpreting scientific texts, and the results are presented in Figure 7.

When comparing AI tools’ global evaluation scores with and without human expertise as a reference, ELISE consistently demonstrated the highest alignment with expert-level responses. For Comprehension, ELISE’s score remained stable (8.32–8.42), whereas other tools demonstrated greater variations: Epsilon (7.56–8.00), ChatGPT (7.48–8.19), SciSpace/Typeset (7.43–7.76) and Humata (6.66 and 6.82). In the Analysis criterion, ELISE also maintained minimal variation (7.98–8.13), outperforming ChatGPT (7.10–7.81), SciSpace/Typeset (7.10–7.52), Epsilon (6.98–7.83) and Humata (6.86–6.87).

Notably, ChatGPT, Epsilon, and SciSpace/Typeset exhibited the largest score increases when human expertise was not used as a reference, with variations ranging from 0.3 to 0.9 points. These results suggest that AI evaluators models might overestimate AI-generate answers with a more lenient AI tools-assessments considering also some of the evaluators (GPT-4o and o1-Preview) self-assess by scoring ChatGPT answers. In contrast, ELISE consistently produced reliable responses, with minimal variation between the two evaluation settings, reinforcing its alignment with expert-level reasoning. Humata followed a similar trend, but with significantly lower scores, indicating less overall accuracy and robustness compared to ELISE.

**Figure 8:**
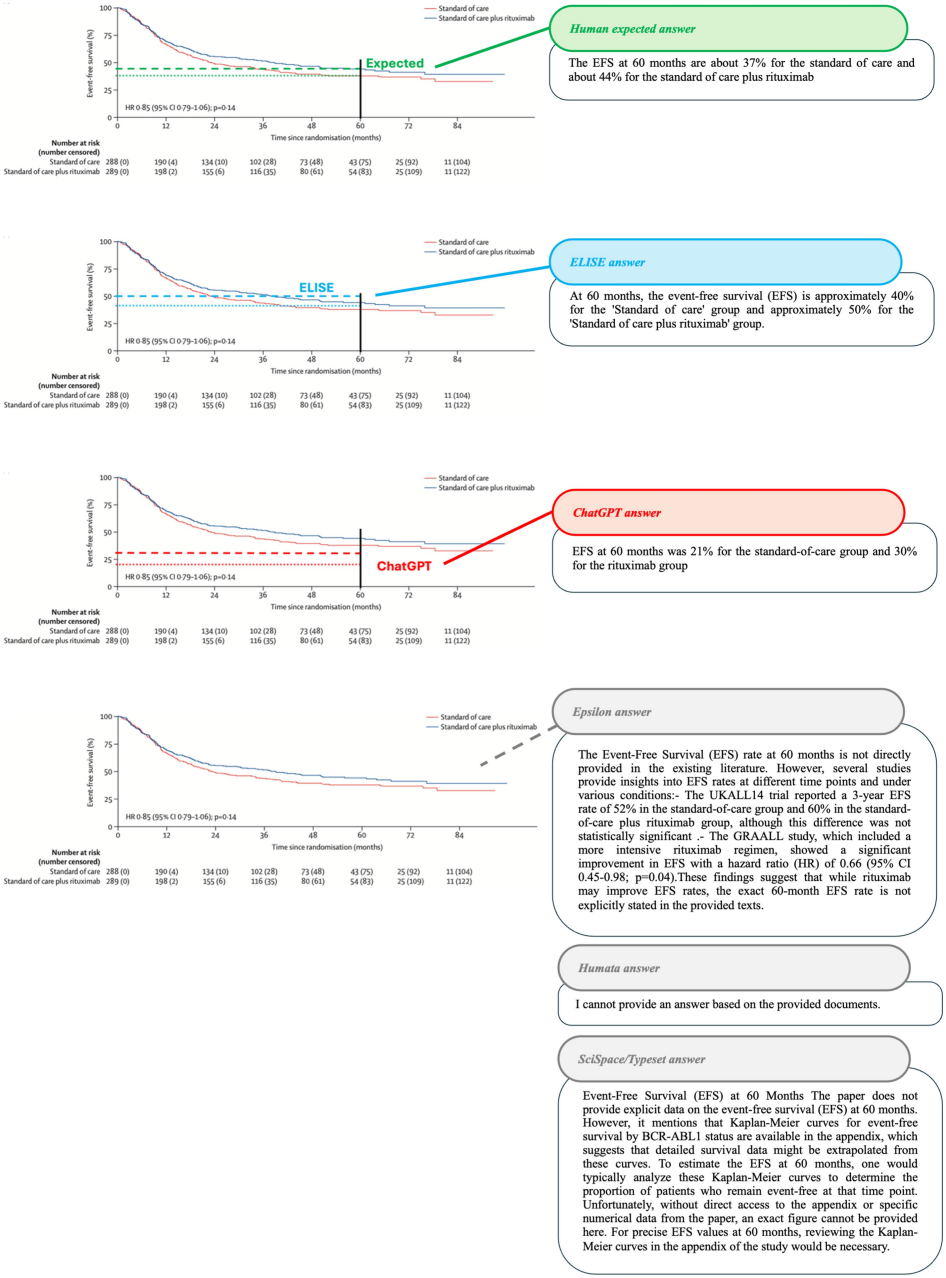
Accuracy in extracting event-free survival (EFS) at 60 months from Article 8 (Marks et al., 2022). Comparison of AI tool responses, with expected values shown in green, ELISE responses in blue, ChatGPT responses in red, and Other AI tools responses in gray (lines and dots) on Article 8 screenshot with the associated outputs of each AI tools.

**Figure 9:**
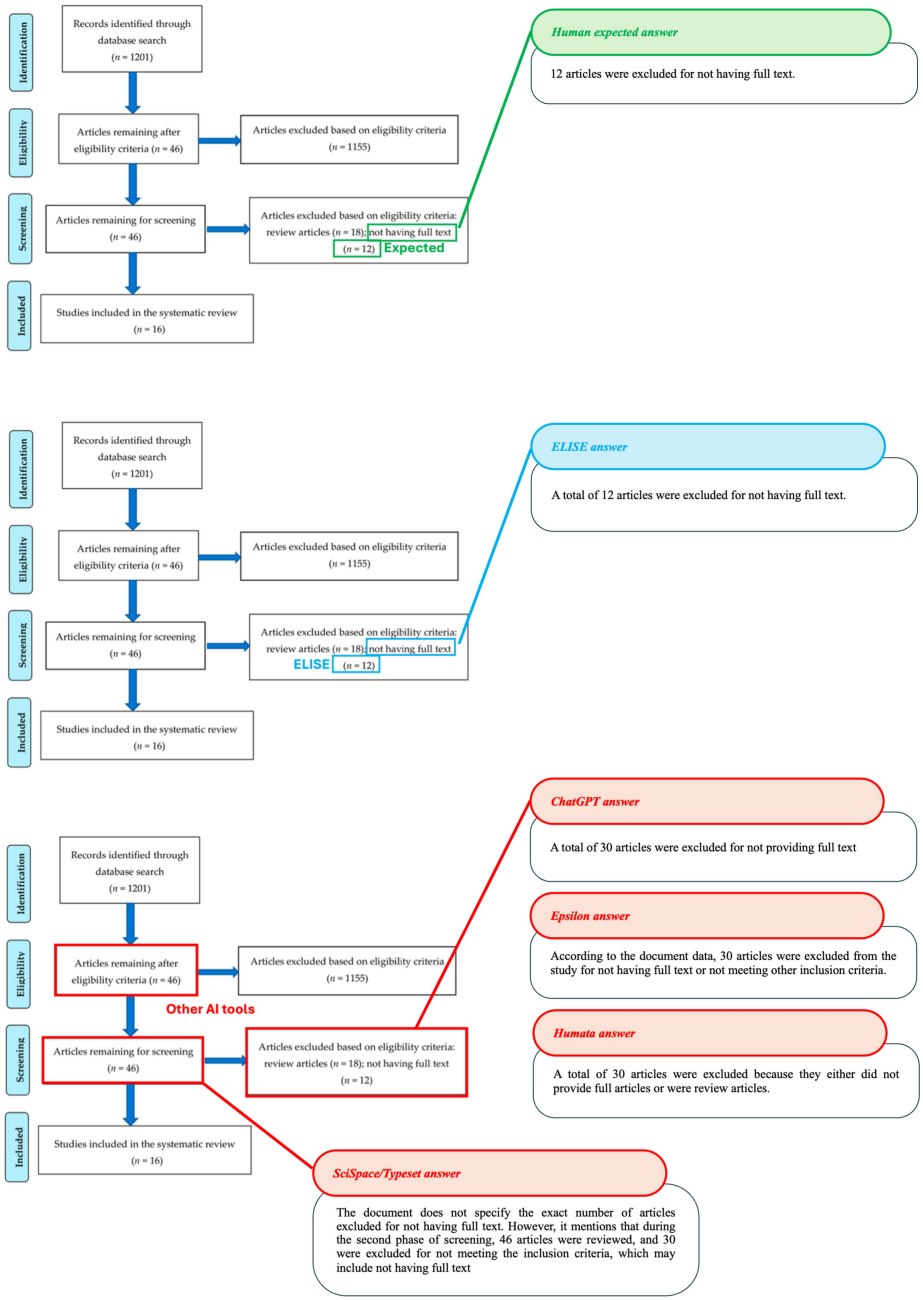
Accuracy in identifying articles excluded for missing full text (Article 5 - Jones et al., 2021). Comparison of AI tool responses to the following question: How many articles were excluded for not having full text? With expected values shown in green, ELISE responses in blue and incorrect Other AI tools responses in red on Article 5 screenshot with the associated outputs of each AI tools.

**Figure 10:**
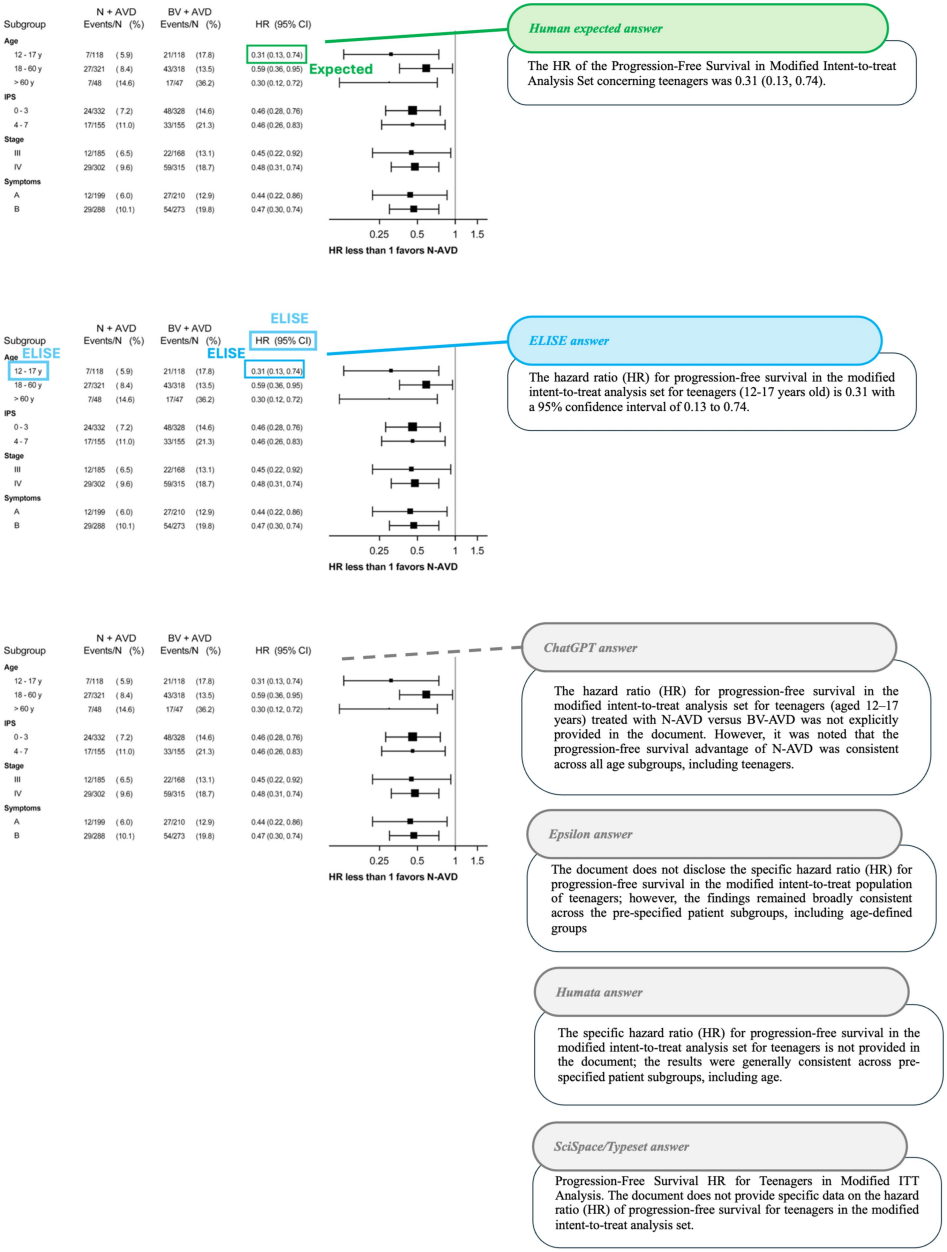
Accuracy in identifying hazard ratios (HR) for progression-free survival (Article 2 - Herrera et al., 2024). Comparison of AI tools responses for the question “What is the HR of the Progression-Free Survival in Modified Intent-to-treat Analysis Set for the teenagers?” with expected values shown in green, ELISE responses in blue and Other AI tools responses in gray on Article 2 screenshot with the associated outputs of each AI tools.

To further validate these observations, specific cases where AI tools exhibited string discrepancies were analyzed. The results are visually represented in Figures 8–10.

In the first case (Figure 8), AI tools were required to extract Event-Free Survival (EFS) percentages at 60 months from a Kaplan–Meier curve. Epsilon, Humata and SciSpace/Typeset failed to generate relevant responses, while ChatGPT provided incorrect values (21 and 30% instead of 37 and 44%). In contrast, ELISE generated the closest approximation (40 and 50%) and explicitly indicated a margin of error, demonstrating a more expert-like approach to data interpretation.

In another example (Figure 9), AI tools had to identify the numbers of excluded articles based on specific selection criteria. While SciSpace/Typeset was unable to provide an exact number, ChatGPT, Epsilon and Humata misinterpret the exclusion criteria, leading to incorrect responses. Only ELISE successfully differentiated between exclusion categories and provided the correct answer (12 articles excluded due to missing full text, demonstrating its superior ability to recognize complex selection criteria and accurately extract relevant numerical data.

The third case (Figure 10) required AI tools to identify a Hazard Ratio (HR) for a specific population within a data table, necessitating both vertical and horizontal reading to locate the expected value. ELISE was the only AI tool capable of retrieving the correct HR (0.31, 95% CI: 0.13–0.74). Moreover, it provided a response even more precise than the expected answer, showcasing its advanced document parsing capabilities and its ability to accurately interpret structured data, a task where all other AI tools failed.

These findings (Figures 7–10 and Supplementary Tables 1–3) reinforce that ELISE is the AI tool that aligns most closely with human expertise across all tested evaluation criteria. Unlike other models, which exhibited greater variability and inconsistencies, ELISE consistently provided responses that matched expert expectations, particularly in challenging tasks involving graph interpretation, inlay detection, and the comprehension of complex scientific data.

### 3.7 Overall results

**Figure 11:**
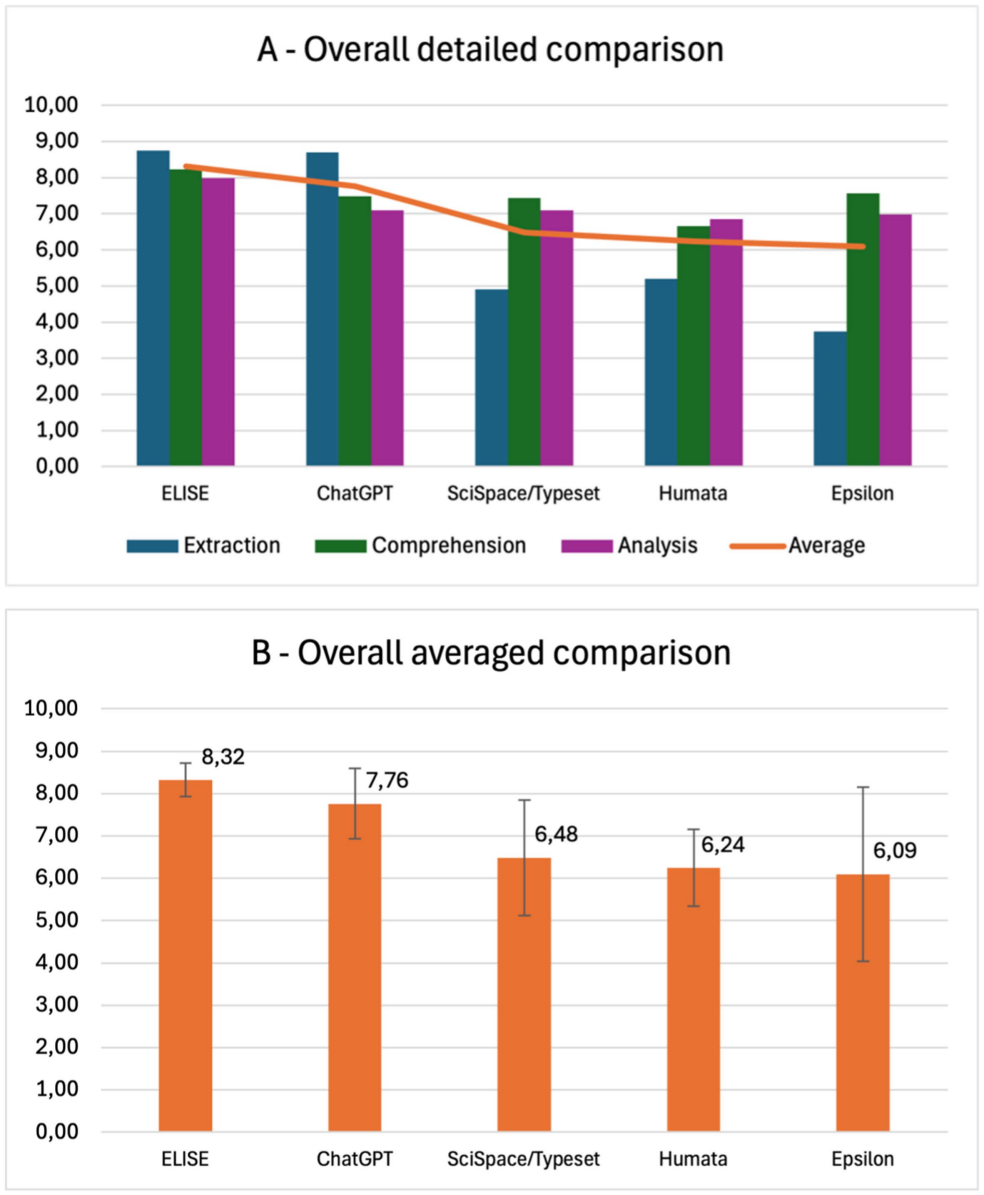
ELISE as the leading AI tool for scientific literature review, from data extraction to accurate analysis. **(A)** Detailed comparison of AI tools (ELISE, ChatGPT, SciSpace/Typeset, Humata, Epsilon) across extraction, comprehension, and analysis criteria. **(B)** Global averaged comparison, confirming ELISE’s superior overall performance.

To provide a comprehensive overview of the AI tools’ evaluation, a detailed comparison of scores across criteria (Figure 11A) and an overall averaged comparison (Figure 11B) were conducted.

The results confirm that ELISE consistently achieved high scores across all criteria, demonstrating minimal variation between Extraction, Comprehension and Analysis.

Among the evaluated tools, ChatGPT performed best in Extraction but exhibited lower efficiency in Comprehension and Analysis, indicating its strength in retrieving structured metadata but its relative weakness in processing and interpreting scientific content. In contrast, SciSpace/Typeset, Humata and Epsilon showed poor performance in Extraction but performed moderately better in Comprehension and Analysis, although their results remained less relevant and less consistent than ELISE’s.

The global average comparison further reinforces these observations. ELISE emerges as the most effective AI tool, followed by ChatGPT, then SciSpace/Typeset, Humata and Epsilon, which obtained similar but lower overall scores. These findings underscore the importance of an AI tool’s ability to handle the entire research workflow, from accurate data extraction to in-depth comprehension and critical analysis, ensuring its reliability for scientific literature processing across various fields and study types.

### 3.8 Statistical analysis

To validate the observed performance differences among AI tools, a comprehensive statistical analysis was conducted. Results for each criterion were included in the global average evaluation (Figures 2F, 4F, 5F).

A unidirectional ANOVA test (*data not shown*) confirmed that AI tool performance varied significantly depending on the evaluation criterion, with some tools excelling in certain task underperforming in others. Additionally, two-way ANOVA tests (*data not shown*) demonstrated that AI tools differed significantly from each other across all criteria, confirming that no single evaluation metric can fully determine an AI tool’s effectiveness in scientific literature analysis.

Interestingly, results indicated no significant interaction effect between AI tools and evaluation criteria, meaning that performance rankings remained consistent regardless of the assessment category. This supports the robustness of the conclusions drawn in this study and validates the methodological soundness of the evaluation framework.

### 3.9 ECACT score

To ensure a rigorous and holistic evaluation framework, an ECACT score was developed, incorporating the Extraction, Comprehension and Analysis criteria alongside two additional dimensions: Compliance and Traceability. These complementary criteria are essential to assessing an AI tool’s reliability and adherence to scientific best practices.

The Compliance criterion evaluates whether an AI tool follows predefined guidelines and exclusively relies on documented content to generate responses. The traceability criterion assesses the tool’s ability to highlight the relevant data sources that were used to generate its answers. These criteria provide a more nuanced understanding of each AI model’s transparency and scientific rigor.

To reflect the importance of each criterion, a weighting system was applied. The Analysis criterion received the highest weight, followed by Comprehension, then Extraction. Similarly, Traceability was weighted equivalently to Analysis, while Compliance was weighted at the same level as Comprehension, ensuring a balanced assessment of AI tools’ capabilities.

**Figure 12:**
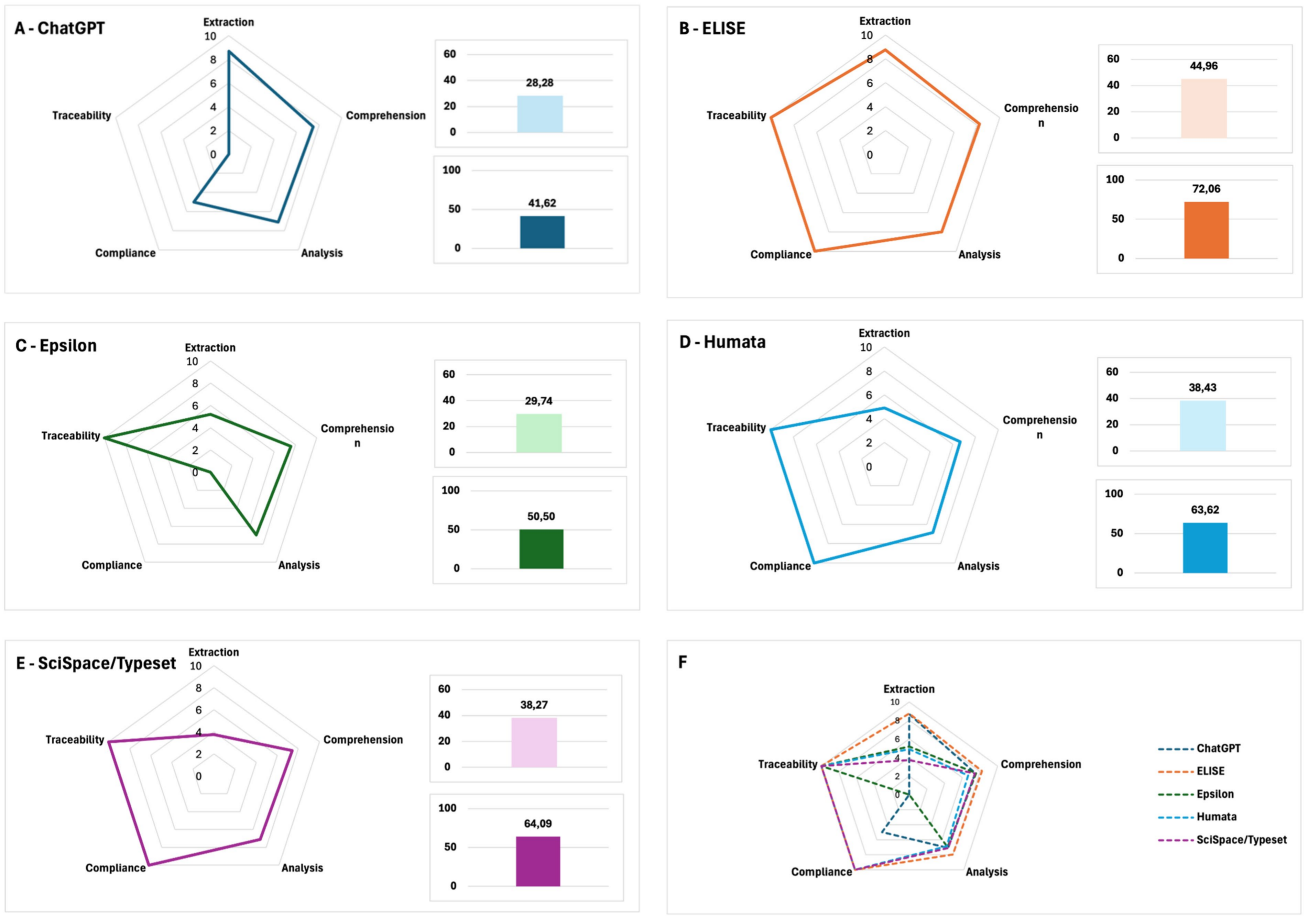
ECACT score evaluation and AI tool comparison. **(A–E)** Radar charts illustrating AI tools’ performance across all evaluation criteria. **(F)** Global ECACT scores, confirming ELISE’s superior reliability and effectiveness across all dimensions.

The results, presented in Figure 12, show that ELISE (Figure 12B) demonstrated the highest performance across all evaluated criteria, achieving both a high global score and a strong ECACT score. In contrast, ChatGPT (Figure 12A) exhibited deficiency in Traceability, while Epsilon (Figure 12C) performed poorly in Compliance and Extraction. Humata and SciSpace/Typeset (Figures 12D,E), despite achieving moderate results in Comprehension and Analysis, struggled significantly in Extraction, limiting their effectiveness in handling end-to-end scientific document processing.

The final ECACT scores were calculated as follows:

- ELISE: Global Score = 44.96, ECACT Score = 72.06.
- ChatGPT: Global Score = 28.28, ECACT Score = 41.62.
- Epsilon: Global Score = 29.74, ECACT Score = 50.50.
- Humata: Global Score = 38.43, ECACT Score = 63.62.
- Scispace/Typeset: Global Score = 38.27, ECACT Score = 64.09.

A significant shift in AI tools rankings was observed when Compliance and Traceability were factored into the evaluation, further reinforcing the need for transparency and guideline adherence in AI-driven research tools.

Notably, ChatGPT’s performance declined significantly due to its lack of traceability, while Epsilon’s overall score decreased due to its failure to comply with evaluation guidelines. Conversely, ELISE remained consistently at the top (Figure 12F), demonstrating that it is not only the most performant AI tool for scientific literature analysis but also the most reliable in terms of transparency and methodological rigor.

### 3.10 Sensitivity analysis of the ECACT score

To assess the robustness of the ECACT framework, a sensitivity analysis was performed by modifying the relative weights of each criterion (e.g., equal weights; Compliance and Traceability prioritized). While minor fluctuations were observed in the ranking of mid-performing tools, the top and bottom positions remained consistent. ELISE systematically outperformed other tools across all tested schemes (see Supplementary Table 2 and Supplementary Figure 4), confirming the resilience of the ECACT methodology.

## 4 Discussion and future directions

AI tools are increasingly transforming workflows in pharmaceuticals, biotechnology, and Medtech by automating data extraction, structuring complex information, and streamlining regulatory and scientific documentation. These tools leverage Named Entity Recognition (NER) to efficiently identify key data points, reducing manual workload and accelerating critical decision-making processes. However, despite these advantages, significant challenges remain, particularly in ensuring the reliability, accuracy, and contextual relevance of AI-generated outputs. Many traditional AI struggle to interpret complex technical content, leading to misinterpretations, inconsistencies, and errors in data extraction. These limitations highlight the need for AI solutions capable of handling industry-specific requirements, where precision, compliance, and traceability are essential for regulatory submissions, clinical trials, and scientific validation.

To address these limitations, the integration of Retrieval-Augmented Generation (RAG) with Large Language Models (LLMs) offers a promising solution for improving accuracy, reliability, and compliance in AI-driven document analysis. By combining advanced retrieval mechanisms with context-aware generation, this approach reduces dependence on pre-trained datasets, which may contain biases or outdated information, and instead ensures fact-based, real-time content generation (Mostafapour et al., 2024; Doyal et al., 2023). One of the most persistent challenges in regulated industries like pharma and Medtech remains the parsing of unstructured documents, including clinical trial reports, regulatory filings, and research publications. In this study, ELISE’s superior performance, closely aligned with human expertise, can be attributed to its advanced parsing capabilities. Unlike other AI tools, ELISE demonstrated a higher efficiency in processing text, formula and table, surpassing industry alternatives such as Megaparse, Llama or Unstructured. Preliminary study demonstrated a Normalized Edit Distance (NED) —how different two elements are by counting the minimum changes (insertions, deletions, substitutions) needed to transform one into the other, normalized by the longest element’s length with 0 as identical and 1 completely different—respectively of 0.558241, 0.622417, 0.574559, and 0.490463 for Megaparse, Llama, Unstructured and Matsu (ELISE parser developed by Biolevate) on the global performance metric for textual content. When combined with context-aware modeling, this feature enhances query interpretation, ensure more precise responses and improve data traceability, making ELISE particularly adapted to regulatory and clinical applications.

The reliability of AI-generated outputs remains a key concern for industries where compliance with regulatory framework is non-negotiable. One striking example from this study (detailed in Supplementary Figure 2) highlights a critical issue: ChatGPT provided a DOI for an article when no other AI tools succeeded, but further investigation revealed that this DOI was not present in the original document, ChatGPT had sourced it externally, violating strict data integrity guidelines. Such discrepancies underscore the importance of traceability and compliance features in AI tools, particularly in clinical research, regulatory submissions, and drug development workflows, where data provenance must be verifiable and reproducible.

To ensure a rigorous and unbiased evaluation of AI tools, a reference human answer was established to benchmark AI-generated responses. To further mitigate evaluation biases, AI Evaluator models were incorporated into the scoring methodology. By leveraging LLMs trained on diverse datasets, these evaluators ensure that responses are analyzed based on factual accuracy rather than subjective human biases. This multi-model validation approach minimizes overestimated AI-generated responses and ensures a more reliable assessment of AI performance, particularly in regulatory and clinical settings.

AI tools such as ChatGPT demonstrate remarkable capabilities in processing large volumes of data, offering significant advantages in speed and accessibility. However, their lack of industry-specific knowledge and contextual awareness makes human oversight essential in critical applications. This study addressed this gap by using expert-defined reference answers, ensuring AI-generated content meets the highest standards of accuracy and relevance for pharmaceutical, biotechnological, and medical applications. Moreover, the ethical and regulatory implications of AI-driven research and documentation must be considered, particularly concerning biases, transparency, and compliance with industry standard. While AI can augment scientific workflow, it cannot replace human expertise. Instead, a hybrid model, where AI supports human decision-making while ensuring data integrity and compliance, offers the most reliable and scalable approach for integrating AI into regulated industries (Mehta et al., 2024; Mostafapour et al., 2024; Ahaley et al., 2024; Singh et al., 2024).

One of the key differentiators among AI tools is compliance and traceability. AI solutions like ELISE integrate built-in compliance mechanisms, allowing for systematic verification of extracted data and response relevance. Unlike general-purpose AI models, which lack transparent methodologies, ELISE explicitly highlights source data and provides traceability on how each response was generated. This feature is critical for regulatory bodies and compliance teams in the pharma, biotech, and Medtech industries, where decision-making must be based on verifiable evidence rather than opaque AI-generated summaries. Furthermore, AI tools capable of explaining their reasoning processes, such as ELISE, allow human experts to refine AI queries, optimize search strategies, and improve model training over time, making them more aligned with human expertise (as demonstrated in Supplementary Figure 3).

Despite advancements in Deep Neural Networks (DNNs), Natural Language Processing (NLP), and Transformers architectures, AI models still struggle with high-level analytical reasoning, contextual variation, and long-form document coherence. These challenges impact critical decision-making in pharmaceutical and medical research, where AI-generated insights must be reliable, interpretable, and reproducible. To address these gaps, state-of-the-art techniques such as Pointer-Generator Networks and Sparse Attention Transformers are being implemented to enhance scientific summarization, improve structured data interpretation, and extract meaningful insights from large-scale regulatory of clinical documents (Tsirmpas et al., 2024).

Given the specialized needs of pharma, biotech, and Medtech, AI tools should be designed with modular adaptability, allowing organizations to select and integrate the most suitable models for their specific applications. As AI becomes more deeply embedded in regulatory, clinical, and research workflows, standardized industry guidelines must be established to ensure transparency, compliance, and ethical AI deployment. Human oversight will continue to play a critical role in refining AI-generated insights, ensuring scientific validity, and maintaining alignment with industry regulations, reinforcing the value of a hybrid AI-human approach in optimizing research and clinical decision-making (Bran et al., 2024; Meyer-Szary et al., 2024). AI tools that provide explainability, such as ELISE, play a crucial role in enhancing human-AI collaboration. By offering transparency on how responses are generated, these tools enable users to understand the AI’s reasoning process, refine their queries for more precise outputs, and iteratively train the model to align more closely with expert-level expectations (as detailed in the Supplementary Figure 3). This capability is particularly valuable in regulated environments such as pharmaceuticals, biotechnology and Medtech, where interpretability, compliance, and continuous model improvement are essential for integrating AI into decision-making workflows.

### 4.1 ECACT score and standardized AI evaluation in regulated industries

The integration of AI tools into pharmaceutical, clinical, and healthcare workflow presents both significant opportunities and operational challenges. While AI optimizes processes such as medical documentation, literature review and regulatory reporting, the variability in AI-generated content quality necessitates a standardized evaluation framework.

Existing regulatory framework, such as SPIRIT-AI and CONSORT-AI, provide essential guidance for AI-driven clinical trials, ensuring transparency, reproducibility, and accountability (McGenity and Treanor, 2021). However, they do not provide standardized methodologies for assessing AI-generated research and regulatory outputs. Similarly, the PRISMA 2020 Checklist, widely used for systematic reviews, lacks specific AI assessment criteria (Page et al., 2021).

To address this gap, this study introduces a dedicated AI evaluation framework based on three core criteria: Extraction, Comprehension, and Analysis, each associated with a structured set of questions. This approach allows progressive assessment from basic data retrieval to advanced contextual analysis, ensuring AI tools are evaluating on their full operational capacity.

Additionally, data transparency in AI-generated response is a critical factor in regulatory compliance. As highlighted in the *Danler* study, AI models must be assessed not only on their accuracy but also on their ability to justify and trace their responses to verifiable sources. The variability in response quality further reinforces the importance of integrating Compliance and Traceability into AI evaluations (Danler et al., 2024).

While ECACT is not a clinical trial reporting guideline like SPIRIT-AI or CONSORT-AI, it complements these frameworks by offering a performance-based evaluation tool specifically for AI-driven document analysis. Future versions of ECACT may integrate harmonization elements with these standards for broader interoperability.

By incorporating these dimensions into the ECACT score, this study demonstrates that AI tools like ELISE, which follow compliance protocols and ensure traceability, significantly outperform models that do not. For instance, Epsilon, which frequently violates guidelines and generates overly verbose responses, was found to be less reliable despite strong comprehension scores. Similarly, ChatGPT’s inability to provide traceable references led to its reassignment from second to last position when these additional criteria were included.

While ChatGPT demonstrates lower performance in traceability and analysis under the ECACT framework, it consistently ranks high in fluency, syntactic clarity, and readability. These traits make it a valuable option for use cases outside regulatory or high-stakes contexts, such as educational summaries, internal research notes, or exploratory drafts. ECACT should thus be interpreted as a scenario-sensitive evaluation tool, guiding tool selection according to task constraints.

Moving forward, the ECACT score should evolve to incorporate additional rating scales to further refine Compliance and Traceability assessments. Moreover, ethical considerations—including data privacy, processing speed, and AI model resource consumption—must be integrated into AI evaluation frameworks. By establishing a standardized, transparent evaluation methodology, as proposed in this study, AI-driven research and regulatory applications can be optimized while ensuring data integrity and ethical compliance (Danler et al., 2024; Wattanapisit et al., 2023; Tangsrivimol et al., 2025).

The current study is limited to life science and biomedical articles. The sample size (*n* = 9) was deliberately kept small to allow for detailed, multi-criteria assessment of each tool’s performance. However, this limited scale restricts the broader generalizability of the results. Future work will focus on validating the ECACT framework on independent datasets, non-English corpora beyond French-English, and diverse scientific domains such as engineering and social sciences, using larger and more heterogeneous article corpora.

Future versions of ECACT may integrate quantitative metrics of semantic similarity (e.g., cosine distance, ROUGE, BERTScore) to complement evaluator-based assessments and further reduce subjectivity in open-ended tasks.

## 5 Conclusion

The study assessed the performance of AI tools in scientific literature analysis, focusing on Extraction, Comprehension, and Analysis criteria while also introducing Compliance and Traceability as critical evaluation dimensions. ELISE emerged as the most effective tool, demonstrating superior performance across all criteria, particularly in data extraction and analytical reasoning, aligning closely with human expertise. ChatGPT exhibited strong efficiency in data retrieval but struggled with deeper comprehension and analysis, limiting its applicability for highly regulated environments. Epsilon, Humata, and SciSpace/Typeset performed moderately, with notable strengths in comprehension but significant weaknesses in structured data extraction, impacting their reliability for complex scientific and regulatory applications.

A key takeaway from this study, is that human oversight remains indispensable in validating AI-generated content, ensuring accuracy, compliance, and contextual relevance, particularly in pharmaceutical, biotechnological, and Medtech applications where data integrity and regulatory adherence are paramount. While AI tools significantly enhance efficiency in literature analysis and knowledge extraction, they must function as augmentative tools rather than standalone solutions.

To address the variability in AI-generated responses and provide a structured evaluation framework, this study introduced the ECACT score, incorporating Extraction, Comprehension, Analysis, Compliance, and Traceability, as key performance indicators. This scoring system ensures that AI tools are assessed not only for their ability to process scientific content but also for their transparency, adherence to guidelines, and ability to justify their outputs. Moving forward, establishing standardized evaluation frameworks such as ECACT will be crucial for integrating AI-driven solutions into research, clinical, and regulatory environments, ensuring that these tools meet the highest standards of scientific rigor, reliability, and ethical compliance.

## Data Availability

The original contributions presented in the study are publicly available. This data can be found here: https://github.com/Biolevate/SL-EVAL-ECACT.

## Footnotes

^1^ https://github.com/Biolevate/SL-EVAL-ECACT
