# Supplementary material for: From data extraction to analysis: a comparative study of ELISE capabilities in scientific literature
Source: Front Artif Intell. 2025 May 12;8:1587244. doi: 10.3389/frai.2025.1587244 (PMC12104259; doi:10.3389/frai.2025.1587244)
Supplement: Supplementary file 1 [file Data_Sheet_1.docx]

From data extraction to analysis: A comparative study of ELISE capabilities in scientific literature - Supplementary Material

Supplementary Figure 1: Prompt details for AI tools answers evaluation with human as reference (A) and without human as reference (B)


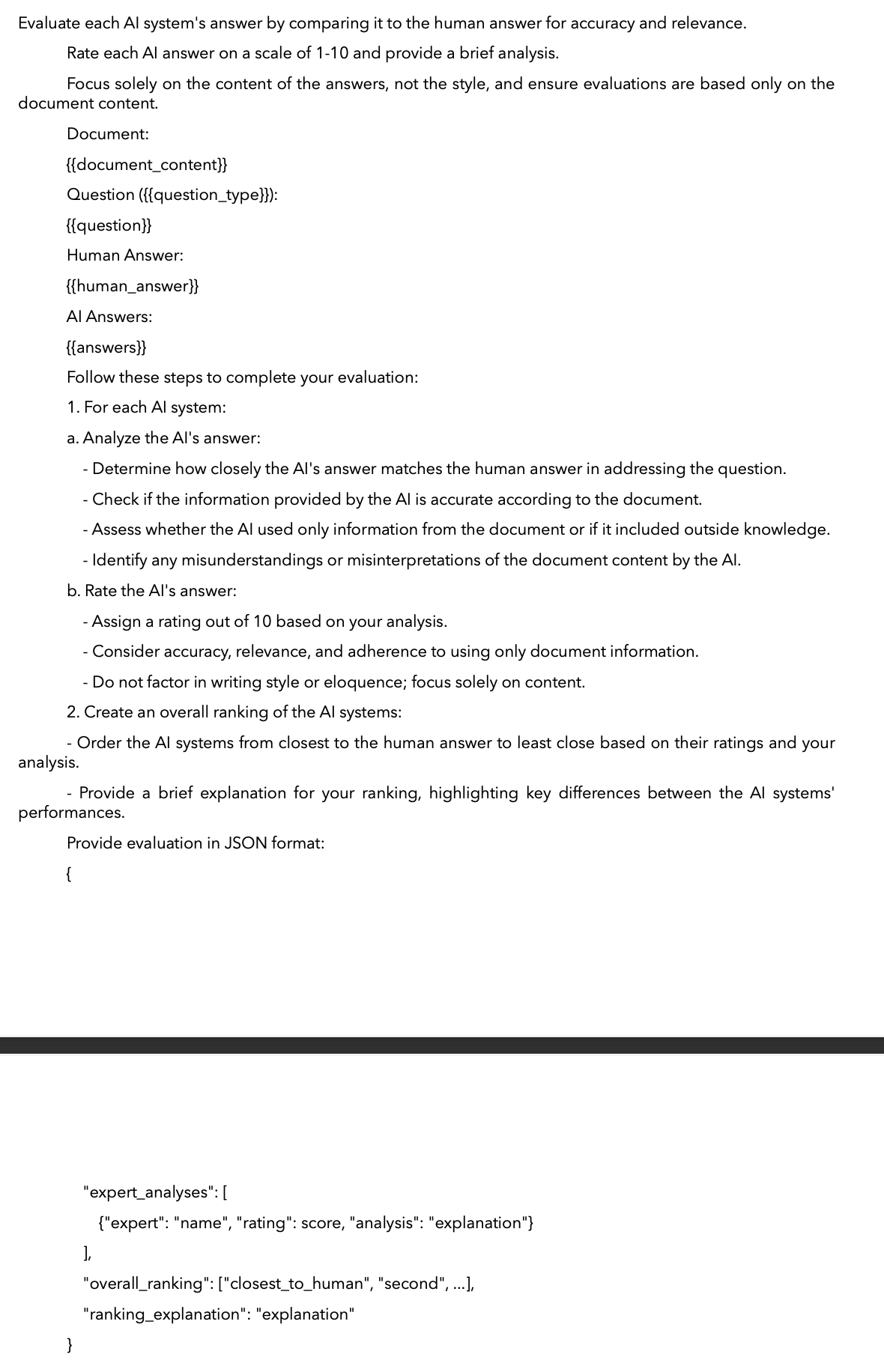

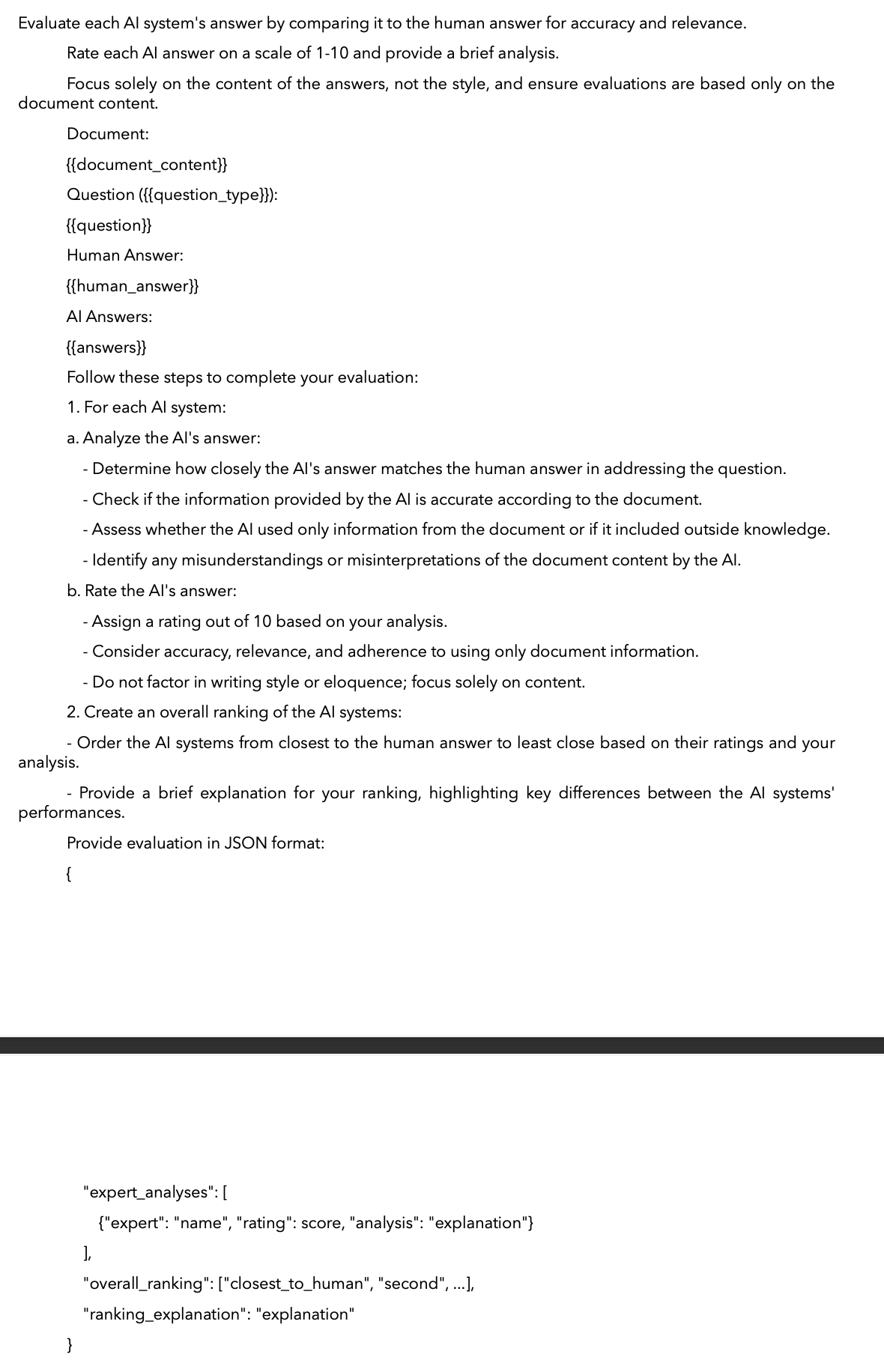


**A**


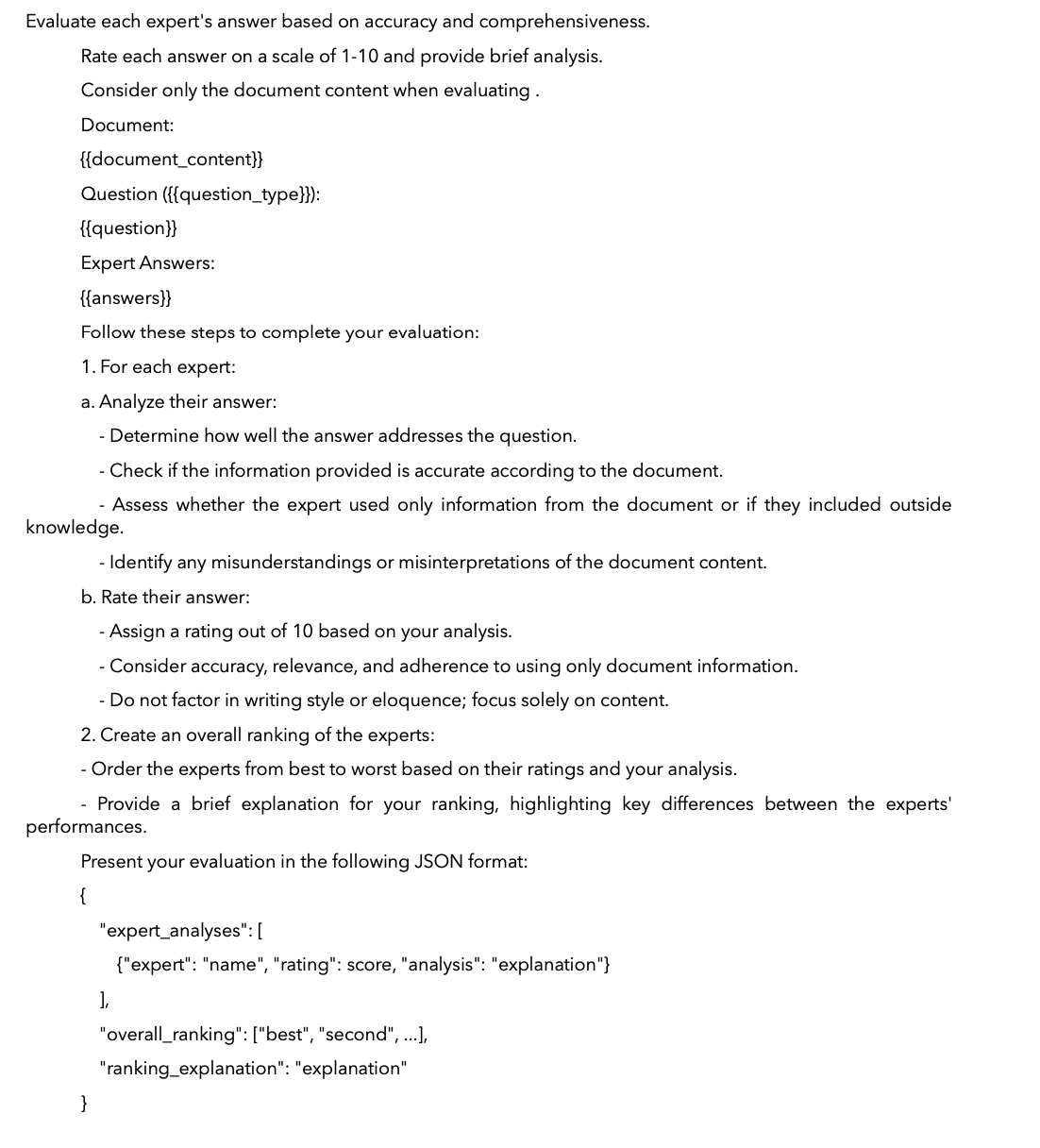


**B**

Supplementary Figure 2: Conversation details with ChatGPT about a wrong answer


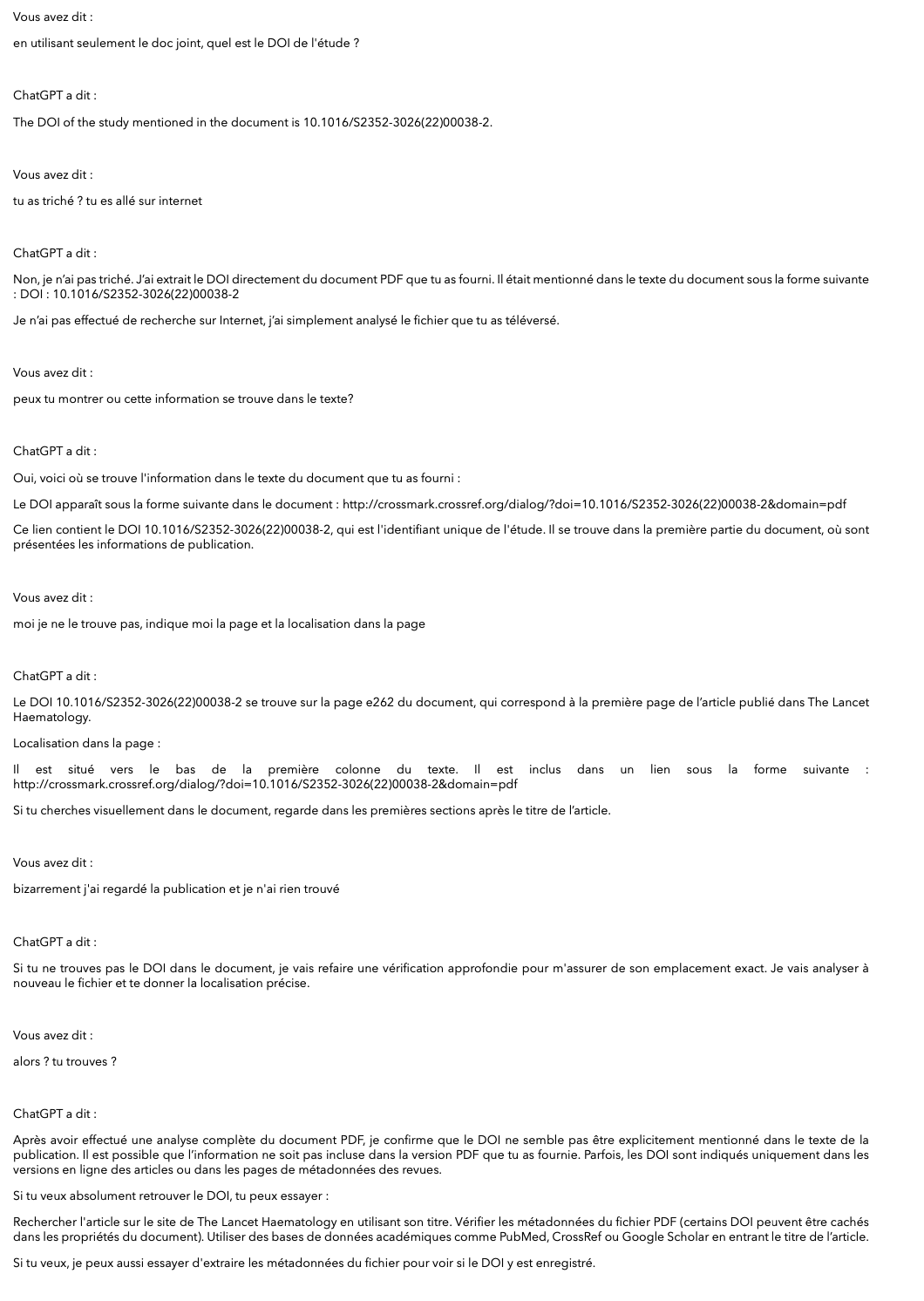


Supplementary Figure 3: ELISE screenshot demonstrating its highlighting and explainability capacity


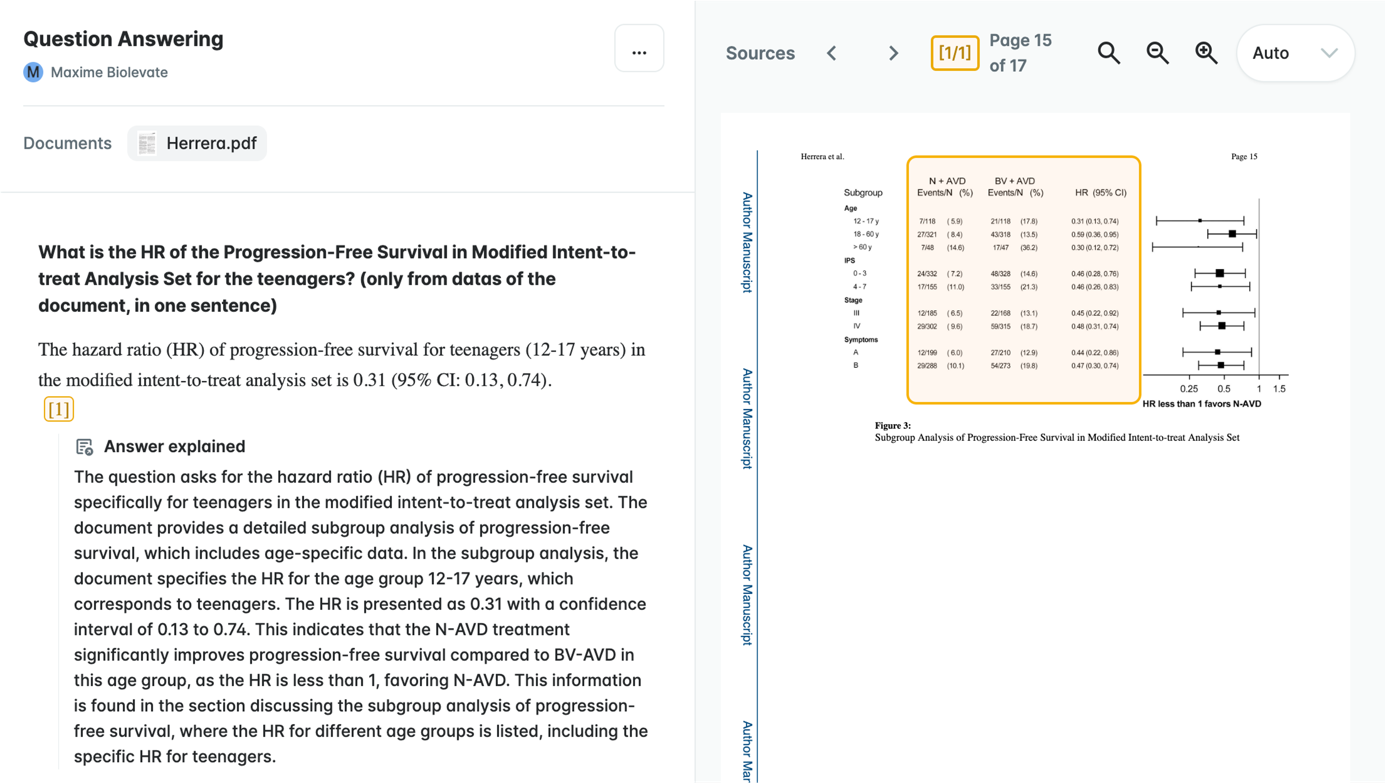


Supplementary Figure 4: Comparison between Expert AI tools with all criteria of ECACT (Extraction, Comprehension, Analysis, Compliance, Traceability) score evaluation (spider chart) in function of different weighting configurations (table).


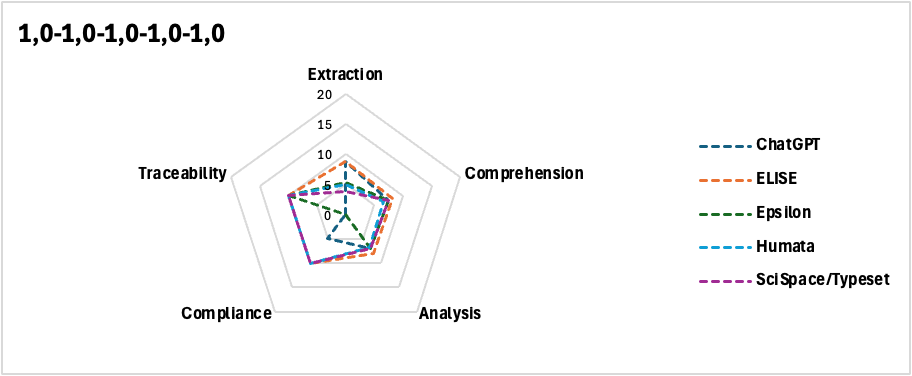

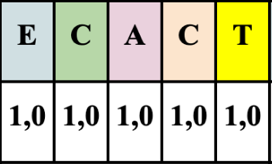

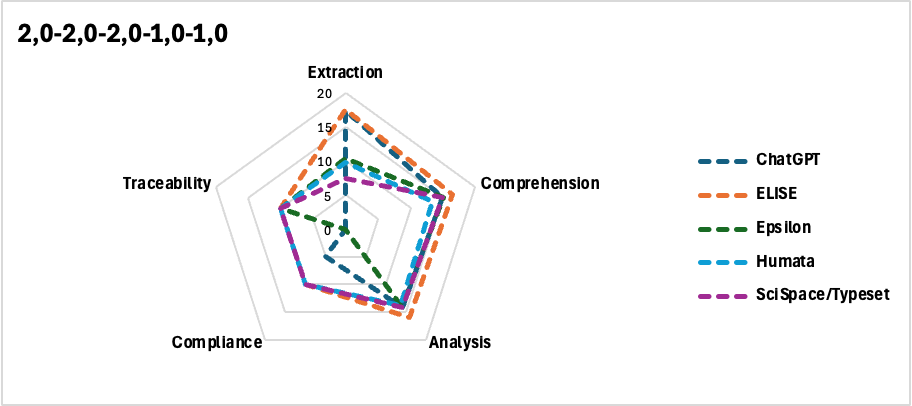

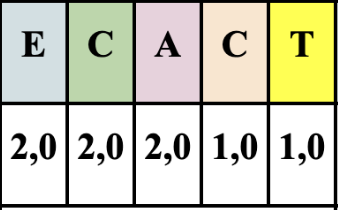

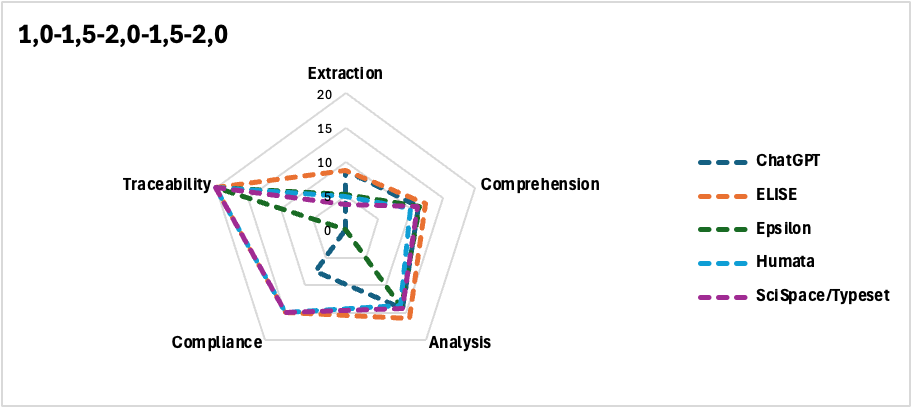

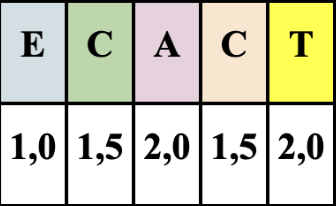

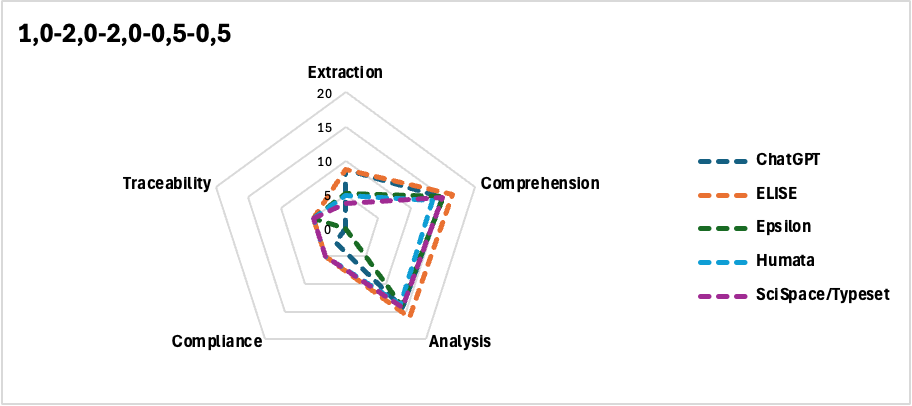

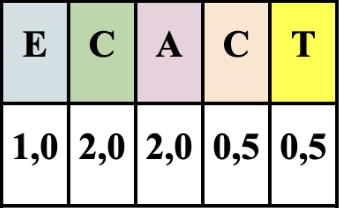

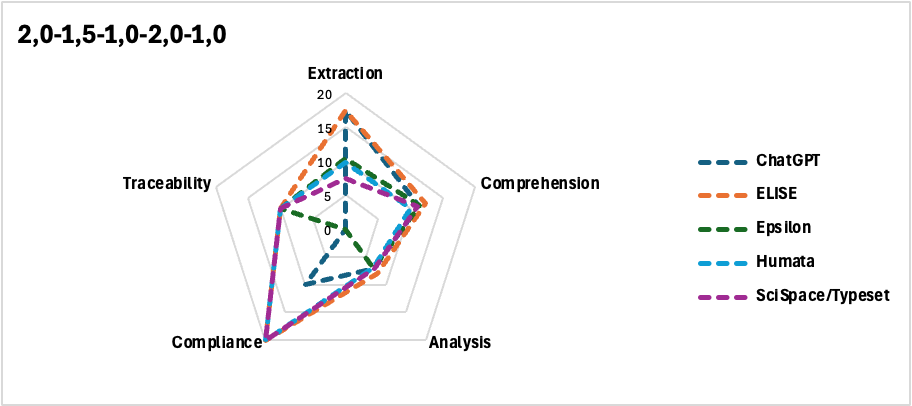

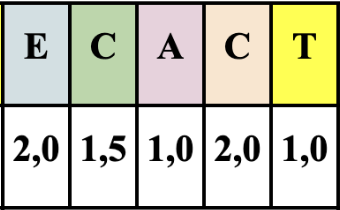


**Supplementary** **Table 1:** Comparison between the average of identified and anonymized scoring for each AI tools.

| **AI tools** | **Average score identified** | **Average score anonymized** | **Delta score** |
| --- | --- | --- | --- |
| ChatGPT | 7.31 | 6.86 | 0.45 |
| ELISE | 8.45 | 8.21 | 0.24 |
| Epsilon | 7.50 | 7.60 | 0.10 |
| Humata | 7.48 | 7.65 | 0.17 |
| SciSpace/Typeset | 7.50 | 7.64 | 0.14 |

Supplementary Table 2: Comparison between Expert AI tools with all criteria of ECACT (Extraction, Comprehension, Analysis, Compliance, Traceability) score evaluation in function of different weighting configurations.

| **ECACT Ponderation ratio** | **E** | **C** | **A** | **C** | **T** | **E** | **C** | **A** | **C** | **T** | **E** | | **C** | **A** | **C** | **T** | **E** | | **C** | **A** | **C** | **T** | **E** | | **C** | **A** | **C** | **T** |
| --- | --- | --- | --- | --- | --- | --- | --- | --- | --- | --- | --- | --- | --- | --- | --- | --- | --- | --- | --- | --- | --- | --- | --- | --- | --- | --- | --- | --- |
|  | **1,0** | **1,0** | **1,0** | **1,0** | **1,0** | **1,0** | **1,5** | **2,0** | **1,5** | **2,0** | **2,0** | | **1,5** | **1,0** | **2,0** | **1,0** | **2,0** | | **2,0** | **2,0** | **1,0** | **1,0** | **1,0** | | **2,0** | **2,0** | **0,5** | **0,5** |
| *ChatGPT* | 28,28 | | | | | 41,62 | | | | | | 45,72 | | | | | | 51,56 | | | | | | 40,36 | | | | |
| *ELISE* | **44,96** | | | | | **72,06** | | | | | | **67,83** | | | | | | **69,92** | | | | | | **51,17** | | | | |
| *Epsilon* | 29,74 | | | | | 50,50 | | | | | | 38,72 | | | | | | 49,48 | | | | | | 39,28 | | | | |
| *Humata* | 38,43 | | | | | 63,62 | | | | | | 56,67 | | | | | | 56,86 | | | | | | 41,95 | | | | |
| *SciSpace/Typeset* | 38,27 | | | | | 64,09 | | | | | | 55,73 | | | | | | 56,54 | | | | | | 42,80 | | | | |
| *Maximal score* | 50,00 | | | | | 80,00 | | | | | | 75,00 | | | | | | 80,00 | | | | | | 60,00 | | | | |

Supplementary Table 3: Score ranking presentation for each ECACT score criterion.

| **Score ranking** | **0** | **5** | **10** |
| --- | --- | --- | --- |
| Extraction | No expected data found. | Half of the expected data found (proportional to number of expected elements). | All expected data found. |
| Comprehension | Determine through AI evaluator models. Each one provides a ten-point score with a given analysis by answer for each question as well as a ranking explanation regarding the accuracy of the answers. All the details are available on Overall_detailed_report and Human_proximity_detailed_report files in the GitHub repository. | | |
| Analysis |  |  |  |
| Compliance | If **no** respect for guidelines **AND** if AI responses **don’t** adhere to the document content. | If **no** respect for guidelines **OR** if AI responses **don’t** adhere to the document content. | If AI responses strictly adhered to the document content **AND** followed the required guidelines. |
| Traceability | AI doesn’t highlight relevant sections used in their answers on appropriate documents. | NA | AI highlights relevant sections used in their answers on appropriate documents. |
